# Development and validation of the mental health service demand and utilization questionnaire

**DOI:** 10.3389/fpubh.2025.1725107

**Published:** 2026-01-12

**Authors:** Shuning Zhang, Siyuan Zhang, Zixuan Feng, Ping Jiang, Yunfeng Gao, Lei Zhang, Kejia Geng, Baojun Wang, Li Duan

**Affiliations:** 1 Department of Health Management Research, Chengde Medical University, Chengde, China; 2 Department of Orthopedic Surgery, Affiliated Hospital of Chengde Medical University, Chengde, China; 3 Department of Medical Affairs, Xidajie Community Health Center, Chengde, China; 4 Department of Medical Affairs, Hebei Mental Health Center/The Sixth People's Hospital of Hebei Province, Baoding, China; 5 Hebei Key Laboratory of Nerve Injury and Repair, Chengde Medical University, Chengde, China

**Keywords:** adolescents, mental health services, older adults, questionnaire development, reliability, validation

## Abstract

**Background:**

As the global burden of mental health issues continues to grow, significant gaps persist in assessing the demand for and utilization of mental health services, particularly among vulnerable populations such as adolescents and older adults. Existing assessment tools often lack cultural and policy relevance, limiting their applicability across diverse social, political, and medical contexts. Guided by a revised Demand and Utilization Framework for Mental Health Services (R-MUSDU), this study aimed to develop and validate a comprehensive Mental Health Service Demand and Utilization Questionnaire (MHSDUQ). The instrument incorporates contextual, individual, and service-related factors to provide a more accurate evaluation of both service needs and patterns of utilization.

**Methods:**

The questionnaire was developed based on the R-MUSDU framework. Initial items were generated through literature clustering analysis (*n* = 4,864), policy document analysis (*n* = 8), and qualitative interviews (*n* = 17), and were subsequently refined via transparent expert consultation (*n* = 18) and a pilot survey (*n* = 60). This study was conducted from May to August 2025 across general hospitals, specialized hospitals, communities, and secondary schools in six provincial-level regions in China. Due to limited data variability in the service utilization section, reliability and validity were assessed specifically for the Service Needs subscale using data from the final sample of 755 participants.

**Results:**

The Service Needs subscale demonstrated high internal consistency (Cronbach’s *α* = 0.975). All items showed significant discriminant values (CR > 3.000, *p* < 0.01) and strong item-total correlations (ranging from 0.765 to 0.914). The exploratory factor analysis (EFA) yielded three factors (attitudinal characteristics, enabling factors, and need factors) comprising 22 items, which collectively accounted for 82.716% of the total variance. Confirmatory factor analysis (CFA) indicated that the modified model fit the data well.

**Conclusion:**

The MHSDUQ demonstrated high validity, internal consistency, and reliability. It is a theory-based and empirically validated tool that can be used to evaluate the mental health service needs and utilization patterns of adolescents and the older adult, demonstrating its potential for application in specific social, cultural, and policy contexts analogous to the study setting.

## Introduction

Mental health issues represent a major public health challenge worldwide, profoundly affecting socioeconomic development and population health (1). According to the Global Burden of Disease study (2), barriers to accessing mental health services are among the leading causes of disability-adjusted life years (DALYs) worldwide. More than one billion people are impacted by mental health conditions (2), which underscore the urgency of public health priority. This challenge is particularly severe in China. A large-scale, nationally representative epidemiological survey indicated that the lifetime prevalence of mental disorders among Chinese adults is 16.6% (3), suggesting that over 230 million people may experience mental health challenges during their lifetime. Vulnerable populations such as adolescents and the older adult are especially at risk. Research indicates that 23.1% of Chinese adolescents exhibit behavioral or emotional concerns (4), while over one-fifth of the older adult population experiences anxiety or depression (5). This high prevalence rates stands in stark contrast to the extremely low utilization of mental health services (6). In high-income countries versus low- and middle-income countries, only one-quarter of individuals with mental disorders have received any form of treatment.

China’s policy-oriented healthcare system has introduced initiatives such as the *National Mental Health Work Plan (2021–2025)* and *Family Doctor Contract Service Policy* aiming to strengthen mental health service delivery, expand community-based care, and improve insurance coverage for mental disorders (7). However, the lack of evidence-based tools to measure implementation effectiveness means that the system still faces significant challenges, including inequitable resource distribution (8), shortages of mental health professionals (9), pervasive social stigma (10).

Andersen’s Behavioral Model of Health Services Use (BMHSU) provides a foundational theoretical framework for understanding and researching mental health service utilization behavior (11). Originating in a Western context, the applicability of its core constructs (predisposing characteristics, enabling resources, and need factors) within unique cultural and policy orientation requires further validation. Consequently, assessment tools derived from this model may lack specificity in capturing culturally and policy-relevant determinants of service utilization, such as familial support, policy directives, and stigma related to mental illness.

Existing assessment tools, such as the World Mental Health Composite International Diagnostic Interview (WMH-CIDI) (12), despite their extensive use in global research, remain insufficient for evaluating mental health service needs and utilization in Chinese regions (13). Globally, multiple scales have been employed to assess mental health service needs. Thornicroft and colleagues developed the Camberwell Assessment of Need (CAN) scale to evaluate perceived needs in domains such as treatment, social support, and daily living functioning (14). The World Mental Health Survey Initiative employed the Service Utilization Questionnaire (SUQ) to quantify the frequency and types of mental health services used across countries (15). Gulliverr et al. (16) developed the Perceived Barriers to Care Questionnaire (PBQ) to identify factors that hinder service utilization. The unique socio-cultural context necessitates context-specific frameworks to understand these disparities. For instance, Anderson’s expanded model (incorporating policy-related variables such as insurance coverage and community service availability) was applied to explain disparities in service utilization in China (17), while a socio-ecological model of mental health service utilization encompassing individual (e.g., stigma), community (e.g., service accessibility), and policy (e.g., funding) factors was also proposed (18).

To address these limitations, the study developed a Mental Health Services Demand and Utilization Questionnaire (MHSDUQ) using a comprehensive, participatory approach. In contrast to previous studies, our instrument integrates evidence from big data literature mining, policy analysis, and direct engagement with stakeholders to ensure cultural, structural, and policy relevance. The questionnaire is designed not only for academic investigation but also for informing practical public health planning and policymaking, particularly in settings where mental health services are undergoing development within a well-defined policy-driven healthcare system. The MHSDUQ aims to provide robust data on unmet needs and service gaps among vulnerable groups, thereby facilitating the development and enhancement of mental health strategies.

## Methods

### Theoretical framework

**Figure 1:**
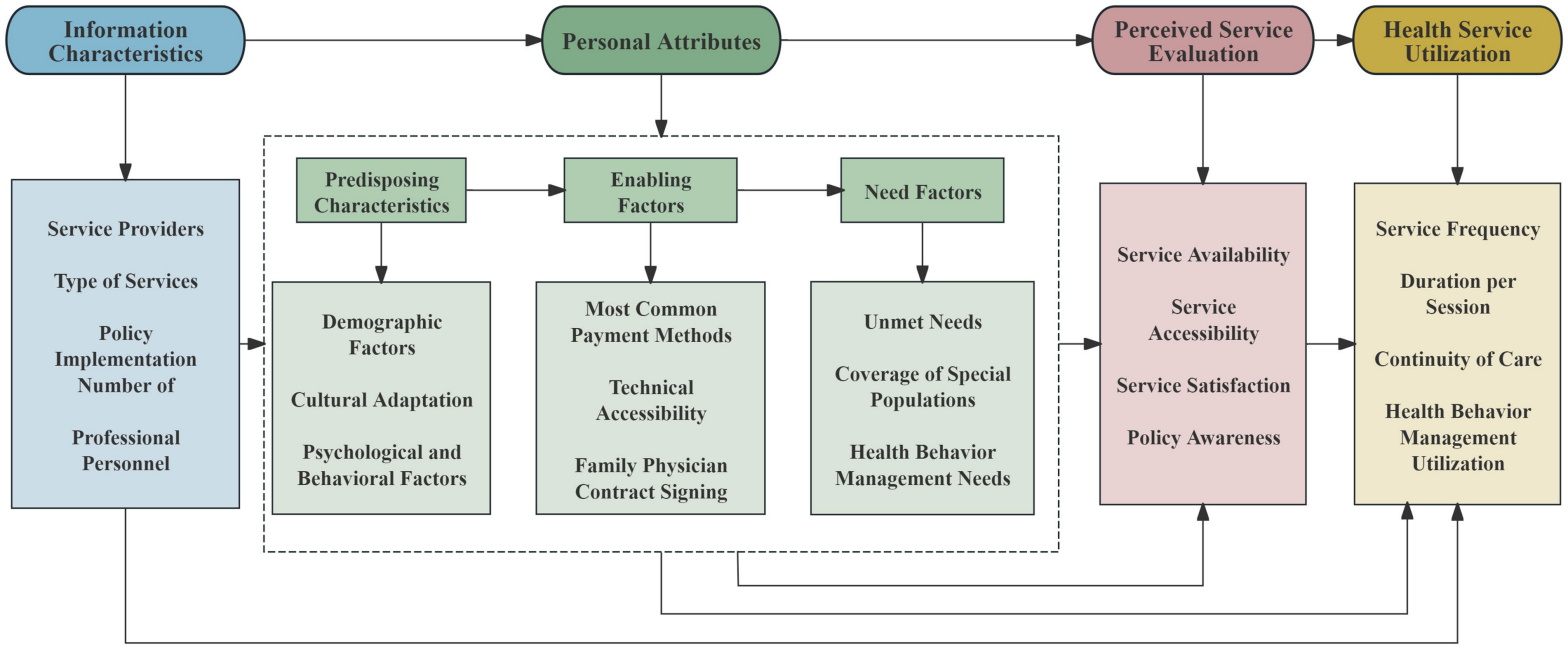
Demand and Utilization Framework for Mental Health Services (R-MUSDU).

BMHSU has evolved considerably since its inception. The most recent and comprehensive version, updated in 2013, adapts the framework to specific contexts (such as complementary and alternative medicine) and vulnerable populations (19). This refined model comprises four core dimensions: contextual characteristics, individual characteristics, health behaviors and outcomes (20). It is widely used to analyze decision-making behaviors and influencing factors related to the acquisition and utilization of health services at the individual or group level, serving as a foundational framework in health services research (21). While retaining the core theoretical structure of the original BMHSU model, this study introduces “policy implementation” into the “contextual characteristics” dimension to reflect the policy-driven approach to healthcare resource allocation in China. Furthermore, the dimensions of “health behaviors” and “health outcomes” in the original model were redefined as “service perception assessment” and “healthcare utilization,” respectively, to align with the research objective of developing a Mental Health Services Demand and Utilization Questionnaire (MHSDUQ).” These adaptations form a logical measurement pathway spanning from the policy environment to individual characteristics and, ultimately, to service utilization outcomes. Figure 1 presents the revised framework, termed the “Demand and Utilization Framework for Mental Health Services (R-MUSDU)” which preserves and enhances the original BMHSU model’s strength in comprehensively analyzing factors—demographic, social, economic and political—that influence individuals’ decisions regarding health service access and use. The R-MUSDU framework directly guided the structure of the MHSDUQ. Specifically, the constructs of “Predisposing Characteristics” and “Enabling Resources” are operationalized as the “Basic Information” section; the “Need factors” correspond to the “Service Needs” subscale; and the “Health Behaviors” dimension is captured by the Service Utilization and Evaluation section.

### Development of the first item pool

#### Phase 1: item generation based on literature clustering analysis

A knowledge mapping analysis of mental health service research was conducted using bibliometric analysis. The specific procedures were as follows: (i) Literature retrieval: Search strategies were developed using a combination of subject headings in the China National Knowledge Infrastructure (CNKI) and Web of Science Core Collection databases (WoSCC). The search period covered from the inception of the CNKI database to March 15, 2025, and from March 15, 2015, to March 15, 2025, for the WoSCC. (ii) Literature download: Retrieved records were saved in text format and imported into CiteSpace 6.3 R1 for filtering and deduplication. After removing duplicates, 4,864 articles were retained for subsequent analyses. (iii) Literature screening: Two researchers independently performed the initial screening “back-to-back.” Final inclusion was determined through discussion based on the predefined eligibility criteria. The inclusion criteria were as follows: for Chinese literature, empirical studies published in “academic journals”; for English literature, articles written in English and classified as “Article.” Conference proceedings, news reports, and irrelevant topics were excluded, as were publications whose full texts could not be obtained. A total of 285 Chinese and 4,579 English articles were included in the analysis. (iv) Scientometric analysis: A keyword co-occurrence clustering analysis was performed using the K-means clustering algorithm to generate knowledge maps of mental health service research domestically and internationally. (v) Item extraction: Fourteen items related to the MHSDUQ were identified and extracted.

The search strategy, keywords clustering results from the bibliometric analysis, along with the potential attributes (theoretical constructs or themes identified from the data), their corresponding levels (operational expressions or value ranges of the attributes), are provided in Supplementary file 1. These potential attributes serve as the foundation for developing the questionnaire items (specific questions formulated to measure these themes).

#### Phase 2: item generation based on policy document analysis

Using keywords such as “mental health,” “psychological health,” and “mental disorders,” we conducted an advanced retrieval of national-level policy databases, including the Chinese Government Web^1^, the National Health Commission of the People’s Republic of China^2^, the Ministry of Civil Affairs of the People’s Republic of China^3^, and the National Healthcare Security Administration^4^. This study aimed to systematically collect policy documents issued by the state regarding the strengthening of mental health service systems. Finally, 9 potential attributes and corresponding levels related to the MHSDUQ were extracted based on bibliometric and clustering analyses, which further scientifically expanded the item pool of the MHSDUQ. The findings are presented in Supplementary file 2.

#### Phase 3: item generation through qualitative interviews

Based on the theoretical framework of R-MUSDU, and incorporating the initially extracted items from the bibliometric analysis and policy document review described above, a preliminary interview outline was developed. Following repeated research team discussions and pre-testing through interviews with six individuals, the guide was revised and finalized. Using purposive sampling, 17 participants were recruited for in-depth qualitative interviews, including adolescents, older adults, and mental health professionals or administrators from settings such as clinical psychiatric departments, primary care community health institutions, middle and high schools, and community-based organizations. Sample size determination followed the principle of thematic saturation in qualitative research (22), whereby recruitment ceased when no new themes emerged from subsequent interviews.

Informed consent was obtained from all participants (and/or their guardians) prior to data collection. Interviews were audio-recorded and supplemented with written notes. The recordings were transcribed verbatim and imported into NVivo 12 for further analysis. Grounded theory methodology was applied, and the data were analyzed through graded coding and categorical analysis based on the revised theoretical framework.

This process generated 11 potential attributes, which were subsequently integrated with the attributes identified in Phase 1 (bibliometric analysis, 19 attributes) and Phase 2 (policy analysis, 9 attributes) to create the preliminary content for the MHSDUQ. The attributes were categorized into 4 key dimensions: contextual characteristics, individual characteristics, service perception assessment, and healthcare utilization. Through thematic integration and deduplication, 30 potential attributes and 143 corresponding levels were extracted and integrated from these three phases, forming a comprehensive theoretical foundation for the questionnaire.

The implementation and analytical results of the qualitative interviews, along with the integrated outcomes from the three phases, are presented in Supplementary file 3.

### Primary questionnaire development

#### Transparent expert consultation

Breaking through the limitations of the traditional Delphi technique, such as its “anonymity” and “iterative feedback,” this study applied a transparent expert consultation (TEC) approach to address these issues. A total of 23 stakeholders were purposively recruited, including 6 experts in health economics policy research/primary health care management, 5 clinical and public health providers (e.g., psychiatrists, general practitioners, and public health physicians), and 6 service users (i.e., healthy individuals, sub-healthy individuals, and diagnosed patients from urban/rural communities or hospitals, comprising 3 adolescents and 3 older adults; 6 corresponding guardians or caregivers were also invited to participate). All participants completed the consultation via online or offline meetings.

The specific procedure was as follows: (i) Prior to the meeting, the research team distributed the first draft of the MHSDUQ to all stakeholders via email or WeChat one week in advance to ensure sufficient familiarity with the research background and the sources of the items. (ii) During the meetings, participants were divided into three groups according to their areas of expertise: A theory group (comprising psychiatrists and health administrators), which reviewed the theoretical coverage and scientific soundness of the existing questionnaire dimensions; A practice group (including physicians, patients, and their caregivers), which evaluated the practicality and applicability of the items; A policy group (involving health economics and policy researchers, health administrators, and physicians), which assessed the alignment of the items with national policies and their potential for future implementation and scalability. (iii) After the meetings, the ratings provided by each group on the importance of the items and dimensions were collected, and medians and inter-quartile ranges (IQR) were calculated. A summary list of revision recommendations was compiled. The following adjustment criteria were applied: items with a median ≥ 4.0 and IQR ≤ 1.0 were retained directly; items with a median ≥ 4.0 but IQR > 1.0 were retained after group discussion; items with a median between 3.0 and 4.0 and IQR ≤ 1.0 were retained after discussion; and items with a median ≤ 3.0, or a median between 3.0 and 4.0 but IQR > 1.0, were deleted.

Based on the expert ratings and discussions, a total of 10 items were revised, including additions, modifications, and deletions. Finally, the second version of the MHSDUQ, comprising 64 items distributed across three preliminary dimensions: Basic Information (22 items), Service Needs (25 items), and Service Utilization and Evaluation (17 items), was developed.

#### Pilot survey

The initial questionnaire was administered to a pilot sample of 60 respondents (30 adolescents and 30 older adults), which was small yet sufficient for conducting item analysis. Among the adolescent participants, 46.67% were female, with a mean age of 16.76 ± 1.76 years. The proportions of those classified as healthy, sub-healthy, and diagnosed with mental disorders were 26.67, 26.67, and 46.67%, respectively. Among the older adult participants, 60.00% were female, with a mean age of (67.47 ± 6.94) years. The proportions of those in the healthy, sub-healthy, and mental disorder categories were 46.67, 33.33, and 20.00%, respectively.

Item analysis showed that the item-total correlation coefficients ranged from 0.736 to 0.927 (*p* < 0.01). Based on feedback from the respondent, items identified as ambiguous or unclear were modified, and the “service utilization” and “service evaluation” sections were merged into a single “service utilization and evaluation” module. Concurrently, based on expert suggestions and participant feedback, 12 new items were generated to cover previously overlooked aspects of service needs and evaluation. The final version of the MHSDUQ consisted of 4 dimensions and 76 items.

The stakeholder composition of the TEC, revisions to the questionnaire based on expert recommendations, and feedback from the pilot survey respondents with corresponding modifications are detailed in Supplementary file 4.

### Formal investigation

#### Participants

A total of 755 participants were successfully recruited for the formal survey. This sample size far exceeds the commonly recommended subject-to-item ratio for factor analysis (e.g., 5:1 to 10:1) and ensures robust statistical power for the planned analyses (23). Data collection took place from May to August 2025, during which adolescents and older adults were recruited as study participants. Questionnaires were distributed through the online WJX platform^5^ and face-to-face methods. Inclusion and exclusion criteria were as follows:

1. Aged 13 ~ 17 or ≥ 60 years, with no restrictions applied regarding gender.
2. Meeting any of the following health status conditions: having no history of mental disorders but requiring preventive mental health services; experiencing mild psychological distress without meeting the diagnostic criteria for mental disorders as defined in the Diagnostic and Statistical Manual of Mental Disorders, Fifth Edition (DSM-5), with a need for preventive or therapeutic mental health services; or meeting DSM-5 diagnostic criteria for a specific mental disorder (e.g., depression, anxiety disorders) and requiring therapeutic mental health services.
3. Possessing the basic interpersonal communication skills necessary to either complete the questionnaire independently or cooperate with researchers to complete it; both the participants (adolescents and older adults) and/or their guardians (for adolescents) provided informed consent to participate in the study.
4. Individuals diagnosed with severe physical illnesses, such as cancer or cardiovascular diseases, and those with severe psychiatric disorders accompanied by hallucinations, aggressive behaviors, or other symptoms that would impede cooperation with the survey were excluded.

**Figure 2:**
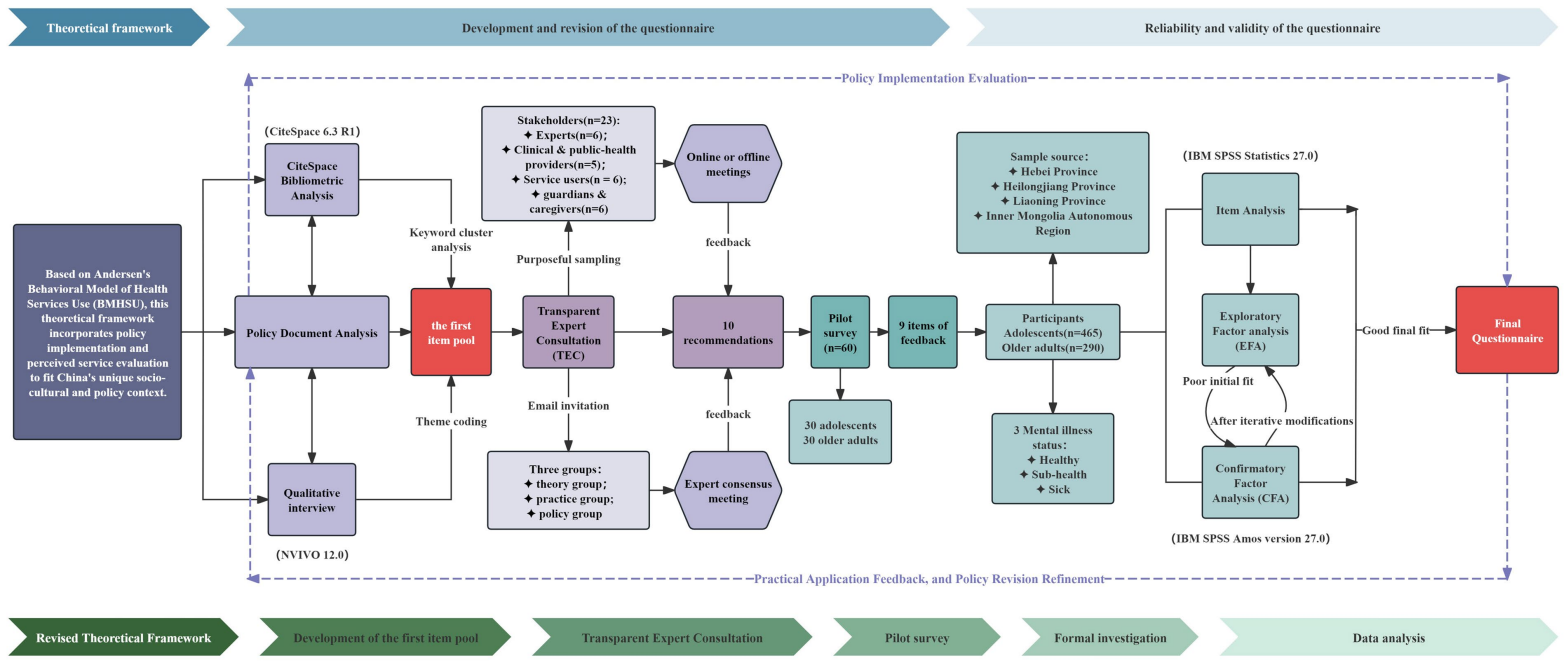
The study design flow chart.

The overall flowchart of the questionnaire development process is presented in Figure 2.

#### Instruments

The resulting version of the MHSDUQ (to be validated) consists of 76 items categorized into three sections. The Basic Information section comprises 22 items collecting demographic and background characteristics. The Service Needs section consists of 25 items, with 24 items using a 5-point Likert scale and one multiple-choice question. The Service Utilization and Evaluation section comprises 29 items, with 27 items assessed on a 6-point scale that includes a “not utilized” option, in addition to two multiple-choice questions.

The mental health status was evaluated with the Chinese Symptom Checklist 90 (SCL-90), a 90 items scale using a five-point Likert scale from 0 to 4 (24). The psychometric properties of the scale have been widely studied with results indicating good validity and reliability (25). The questionnaire was divided into ten factors: somatization, obsessive-compulsive, interpersonal, sensitivity, depression, anxiety, hostility, phobia, and paranoid ideation and psychoticism (26). Scores range from 1 to 5, with different ranges indicating various clinical conditions. A total score exceeding 160 points, over 43 positive items, or any factor score above 2 is considered positive according to national norms (27). In the current study, the SCL-90 demonstrated excellent internal consistency, with a Cronbach’s *α* coefficient of 0.96.

Depressive symptoms were assessed using the Hamilton Rating Scale for Depression (HAMD-17) (28), comprising 8 items rated on a 5-point scale (0–4 points) and 9 items rated on a 3-point scale (0–2 points). A total score above 17 indicates mild to moderate depression, whereas a score below 7 signifies the absence of clinically significant depression. In the present sample, the HAMD-17 showed good internal consistency, with a Cronbach’s *α* coefficient of 0.88.

Anxiety symptoms were evaluated with the Hamilton Anxiety Scale (HAMA-14) (29), consisting of 14 items, each rated on a 5-point scale ranging from 0 to 4. A total score above 14 suggests the presence of clinically significant anxiety. In this study, the HAMA-14 exhibited good internal consistency, with a Cronbach’s α coefficient of 0.87.

#### Data collection and recruitment procedures

Data collection for this study commenced in May 2025 using both online and offline questionnaire distribution methods. Recruitment in general and specialized hospitals was conducted after obtaining approval from the Institutional Research Committee. Psychiatrists identified eligible adolescents and older adults during clinical consultations through diagnostic interviews and physical examination. Participants who met the inclusion criteria were invited to participate. After providing informed consent, the participants completed the questionnaire on-site. In community healthcare settings, recruitment was facilitated within the context of China’s national “Family Doctor Contract Service” policy, in which general practitioners provide basic medical care, public health services, and health management to the registered residents. Using a convenience sampling method, general practitioners assisted the research team in identifying eligible participants (adolescents and older adults), who completed the questionnaires on-site. In secondary schools, the research team coordinated with school principals to recruit participants. Students from junior and senior high schools were selected using random sampling. Homeroom teachers, who were trained uniformly to ensure consistent implementation, assisted in explaining the study to parents and students. Questionnaires were administered both on-site (within the school) and online (outside school).

Throughout the recruitment process, the research team provided detailed explanations of the study background, objectives, inclusion and exclusion criteria, and sample size requirements to institutional administrators, healthcare providers, and potential participants. For online surveys, in addition to distributing the questionnaire link, electronic informed consent forms and contact information of the research team were also provided. To ensure data authenticity, Internet Protocol (IP) addresses were controlled to prevent duplicate responses, and completion time was limited to 40 min. Each submitted questionnaire underwent quality review. Throughout the study, anonymity and confidentiality were strictly maintained.

### Data analysis

IBM SPSS version 27.0 and AMOS 27.0 were used for data analyses. Missing values were processed in SPSS utilizing the series mean imputation approach, which was located under the Replace Missing Values option in the Transform menu. All applicable variables were included in this procedure. Following the imputation, the dataset was verified to contain no remaining missing values. Descriptive statistics are reported as mean and standard deviation (SD) for continuous variables, and as frequency counts and percentages for categorical variables.

Reliability and validity assessments were primarily conducted for the Service Needs subscale. The characteristically low utilization rates of grassroots mental health services among the Chinese population led to insufficient variability and limited valid data in the “Service Utilization and Evaluation” section for robust factor analysis. Consequently, EFA and CFA were conducted exclusively on the 24 single-choice items of the Service Needs subscale to validate its dimensional structure. The entire MHSDUQ, comprising 76 items, was retained for comprehensive assessment, with the other sections serving as crucial descriptive and profiling tools.

Item analysis was performed using four methods: the discrete trend method (standard deviation > 0.80), critical ratio (CR) method (CR > 3.0 with *p* < 0.05), correlation coefficient method (item-total correlation > 0.40), and Cronbach’s alpha if item deleted (CAID; item should not increase *α*). A significance level of *p* < 0.05 was used for all statistical tests.

Reliability was assessed through internal consistency. Internal consistency was evaluated using Cronbach’s α coefficient for the Service Needs subscale and its sub-dimensions. Cronbach’s α values greater than 0.700, 0.800, and 0.900 were considered indicative of acceptable, good, and excellent internal consistency, respectively (30).

Construct validity was assessed using exploratory factor analysis (EFA) and confirmatory factor analyses (EFA and CFA, respectively). The total sample (*n* = 755) was randomly split into two independent subsamples: one for Exploratory Factor Analysis (EFA, *n* = 378) and the other for Confirmatory Factor Analysis (CFA, *n* = 377). EFA was performed using principal component analysis with varimax rotation. The Kaiser-Meyer-Olkin (KMO) measure and Bartlett’s test of sphericity were used to assess the suitability of the data for factor analysis. Factors with eigenvalues greater than 1 were retained for analysis. Items with factor loadings less than 0.40 or with cross-loading differences less than 0.20 were considered for removal. CFA was conducted using maximum likelihood estimation. Model fit was evaluated using the following indices: *χ*^2^/*df* (acceptable if < 5.0), root mean square error of approximation (RMSEA; acceptable if < 0.08), comparative fit index (CFI, acceptable if > 0.90), Tucker-Lewis’s index (TLI, acceptable if > 0.90), and incremental fit index (IFI, acceptable if > 0.90) (31, 32).

## Results

### Demographic characteristics of participants

A total of 755 participants were included in this study, comprising 465 adolescents and 290 older adults. Participants were recruited from six provincial-level administrative regions in China, with most being from Hebei (60.2%), Heilongjiang (19.5%), and Liaoning (12.8%) Provinces. Among the adolescent group (*n* = 465), 249 (53.5%) were junior high school students with a mean age of 12.77 ± 0.82 years, and 216 (46.5%) were senior high school students with an average age of 16.59 ± 0.92 years. The older adult group (*n* = 290) had a mean age of 67.69 ± 14.49 years. Further demographic data for the entire sample (*n* = 755) are shown in Table 1.

**Table 1:** Demographic characteristics of participants (*n* = 755).

Variables	Category	Adolescents (*n* = 465)	Older adults (*n* = 290)
		Means ± SD (±s)/*n* (%)	Means ± SD (±s)/*n* (%)
Mental health status	Healthy	196 (42.2)	131 (45.2)
	Sub-healthy	138 (29.7)	92 (31.7)
	With mental disorder	131 (28.2)	67 (23.1)
Gender	Male	237 (51.0)	139 (47.9)
	Female	228 (49.0)	151 (52.1)
Age	13 ~ 17	16.57 ± 1.70	—
	60 ~ 70	—	65.23 ± 2.78
	70 ~ 80	—	74.44 ± 2.83
	≥80	—	83.73 ± 3.31
Ethnicity	Han	373 (80.2)	219 (75.5)
	Ethnic minorities	92 (19.8)	71 (24.5)
Residence	Urban	194 (41.7)	218 (75.2)
	Rural	271 (58.3)	72 (24.8)
Payment method for healthcare	Urban Employee Basic Medical Insurance	0 (0.0)	116 (40.0)
	Urban and Rural Resident Basic Medical Insurance	175 (37.6)	151 (52.1)
	Medical Assistance	7 (1.5)	0 (0.0)
	Civil Servant Medical Subsidy	6 (1.3)	2 (0.7)
	Commercial Health Insurance	7 (1.5)	0 (0.0)
	Out-of-pocket payment	14 (3.0)	21 (7.2)
	Not Applicable	256 (55.1)	0 (0.0)
Marital status	Unmarried	465 (100.0)	13 (4.5)
	Married	0 (0.0)	241 (83.1)
	Widowed	0 (0.0)	10 (3.4)
	Divorced	0 (0.0)	4 (1.4)
	Not specified	0 (0.0)	22 (7.6)
Monthly household income(USD)	<399	57 (12.3)	19 (6.6)
	400–699	142 (30.5)	41 (14.1)
	700 ~ 1,399	186 (40.0)	151 (52.1)
	≥1,400	80 (17.2)	79 (27.2)
Contracted family doctor services	Yes	13 (2.8)	90 (31.0)
	No	452 (97.2)	200 (69.0)
Sought mental health services previously	Yes	58 (12.5)	13 (4.5)
	No	407 (87.5)	277 (95.5)
Diagnosed with mental disorder (self-report)	Yes	71 (15.3)	111 (38.3)
	No	394 (84.7)	179 (61.7)
Family history of mental disorders	Yes	14 (3.0)	6 (2.1)
	No	451 (97.0)	284 (97.9)

—, indicates not applicable.

During data collection and entry, a large number of missing values in the “Service Utilization and Evaluation” section limited data availability for factor analysis. Therefore, validation was only done for the 24 items of the Service Needs subscale. The sections on service utilization and assessment mainly serve descriptive and categorization purposes, aligning with the identified needs in the service needs subscale.

### Item analysis

Item analysis was conducted using standard deviation, critical ratio (CR), item-total correlation, and Cronbach’s *α* if item deleted (CAID). The standard deviations of all items ranged from 0.993–1.325. All items showed significant CR values (CR > 3.000, *p* < 0.05) and item-total correlations above 0.40. One item (A3) was removed due to an excessively high correlation with another item A4 (*r* = 0.859). The corrected item-total correlations (CITC) ranged from 0.765 to 0.914, and the Cronbach’s *α* coefficients decreased upon deletion of any item, supporting the retention of all remaining items. Detailed results are presented in Table 2.

**Table 2:** Item analysis results.

Item	Standard deviation method		Critical ratio method		Correlation analysis method			Cronbach’s α coefficient method	
	Mean	SD	CR	*p*-value	Item-total correlation	Max inter-item correlation	Item with max correlation	CITC	CAID
A1	2.16	1.173	27.138	0.000	0.717	0.828	A2	0.813	0.757
A2	2.06	1.135	30.178	0.000	0.786	0.840	A3	0.889	0.756
A3	2.16	1.184	34.739	0.000	0.787	0.859*	A4	0.891	0.755
A4	2.20	1.214	34.030	0.000	0.791	0.859*	A3	0.889	0.755
A5	2.20	1.201	35.459	0.000	0.780	0.838	A4	0.884	0.755
A6	2.10	1.107	33.661	0.000	0.642	0.838	A4	0.914	0.756
A7	2.06	1.120	31.708	0.000	0.689	0.842	A8	0.883	0.756
A8	2.07	1.104	29.914	0.000	0.702	0.842	A7	0.886	0.756
A9	2.07	1.117	32.120	0.000	0.723	0.819	A8	0.893	0.756
A10	1.97	0.993	24.403	0.000	0.698	0.759	A9	0.838	0.758
A11	2.11	1.098	29.565	0.000	0.756	0.810	A12	0.866	0.756
A12	2.06	1.044	32.152	0.000	0.742	0.810	A11	0.877	0.757
A13	2.13	1.072	35.053	0.000	0.812	0.807	A12	0.888	0.756
B14	2.38	1.325	32.163	0.000	0.798	0.811	B15	0.803	0.755
B15	2.39	1.272	32.967	0.000	0.785	0.811	B14	0.781	0.756
B16	2.36	1.264	33.927	0.000	0.802	0.807	B14	0.772	0.756
B17	2.12	1.148	28.195	0.000	0.765	0.764	B16	0.765	0.757
C18	2.09	1.102	32.637	0.000	0.758	0.780	A6	0.882	0.756
C19	1.86	1.005	23.098	0.000	0.821	0.806	C21	0.785	0.758
C20	2.03	1.089	28.431	0.000	0.815	0.784	C21	0.814	0.757
C21	1.93	1.024	30.259	0.000	0.792	0.809	C22	0.850	0.757
C22	1.87	1.048	24.920	0.000	0.788	0.809	C21	0.823	0.758
C23	2.00	1.139	28.842	0.000	0.776	0.797	C24	0.828	0.757
C24	2.02	1.090	30.476	0.000	0.765	0.797	C23	0.840	0.757
Retention criteria	<1.5 & > 4.5	≤0.8	<3.000	≥0.05	<0.40 & > 0.85			<0.40	Significant improvement in α coefficient

SD, standard deviation; CR, critical ratio; CITC, corrected item-total correlation; CAID, Cronbach’s alpha if item deleted. *Consider deleting the item.

### Reliability analysis

The internal consistency of the Service Needs subscale was excellent, with a Cronbach’s α coefficient of 0.976 for the total scale. The dimensions also demonstrated good to excellent reliability, with Cronbach’s α values of 0.968 (Predisposing Characteristics), 0.863 (Enabling Factors), and 0.941 (Need Factors), respectively, indicating adequate internal consistency. Additionally, the split-half reliability, as measured by the Spearman-Brown coefficient, was 0.944 for the total scale, further supporting its reliability. Detailed reliability coefficients for each dimension are presented in Table 3.

**Table 3:** Reliability coefficients.

Dimension	Cronbach’s α	Split-half reliability (Spearman-Brown)
Predisposing characteristics	0.968	0.965
Enabling factors	0.863	0.935
Need factors	0.941	0.951
Total subscale	0.976	0.944

### Construct validity analysis

In EFA, the KMO measure was 0.975, and Bartlett’s test of sphericity was significant (*χ*^2^ = 14565.127, *p* < 0.001), indicating that the data were suitable for EFA. Principal component analysis with varimax rotation extracted three factors with eigenvalues greater than 1, accounting for 82.716% of the total variance. Items with factor loadings below 0.40 or cross-loading differences less than 0.2 were considered for removal. Items A13 and C18 cross-loaded on two factors; after discussion, A13 was removed and C18 was retained. The final EFA supported a three-factor structure comprising 22 items, with all factor loadings exceeding 0.40. Items B11 and B12 were reallocated from “Enabling Factors” to “Predisposing Characteristics” based on the factor structure. The rotated component matrix is presented in Table 4.

**Table 4:** Rotated factor loading from exploratory factor analysis (*n* = 755).

Items	Components		
	1	2	3
A1	**0.726**	0.309	0.327
A2	**0.740**	0.399	0.341
A4	**0.798**	0.332	0.350
A5	**0.773**	0.377	0.325
A6	**0.780**	0.435	0.310
A7	**0.732**	0.476	0.270
A8	**0.756**	0.449	0.277
A9	**0.730**	0.432	0.348
A10	**0.648**	0.464	0.308
A11	**0.746**	0.340	0.380
B12	**0.646**	0.480	0.376
B13	**0.601**	0.505	0.425
B14	0.412	0.289	**0.780**
B15	0.397	0.245	**0.806**
B16	0.334	0.280	**0.838**
B17	0.269	0.412	**0.759**
C18	0.537	**0.652**	0.331
C19	0.310	**0.784**	0.295
C20	0.406	**0.736**	0.272
C21	0.398	**0.806**	0.275
C22	0.404	**0.787**	0.237
C23	0.424	**0.728**	0.284
C24	0.399	**0.736**	0.333

Item A3 was deleted based on the results of the item analysis. The bold values indicate the highest absolute factor loading for each item, denoting its assignment to the primary factor.

**Figure 3:**
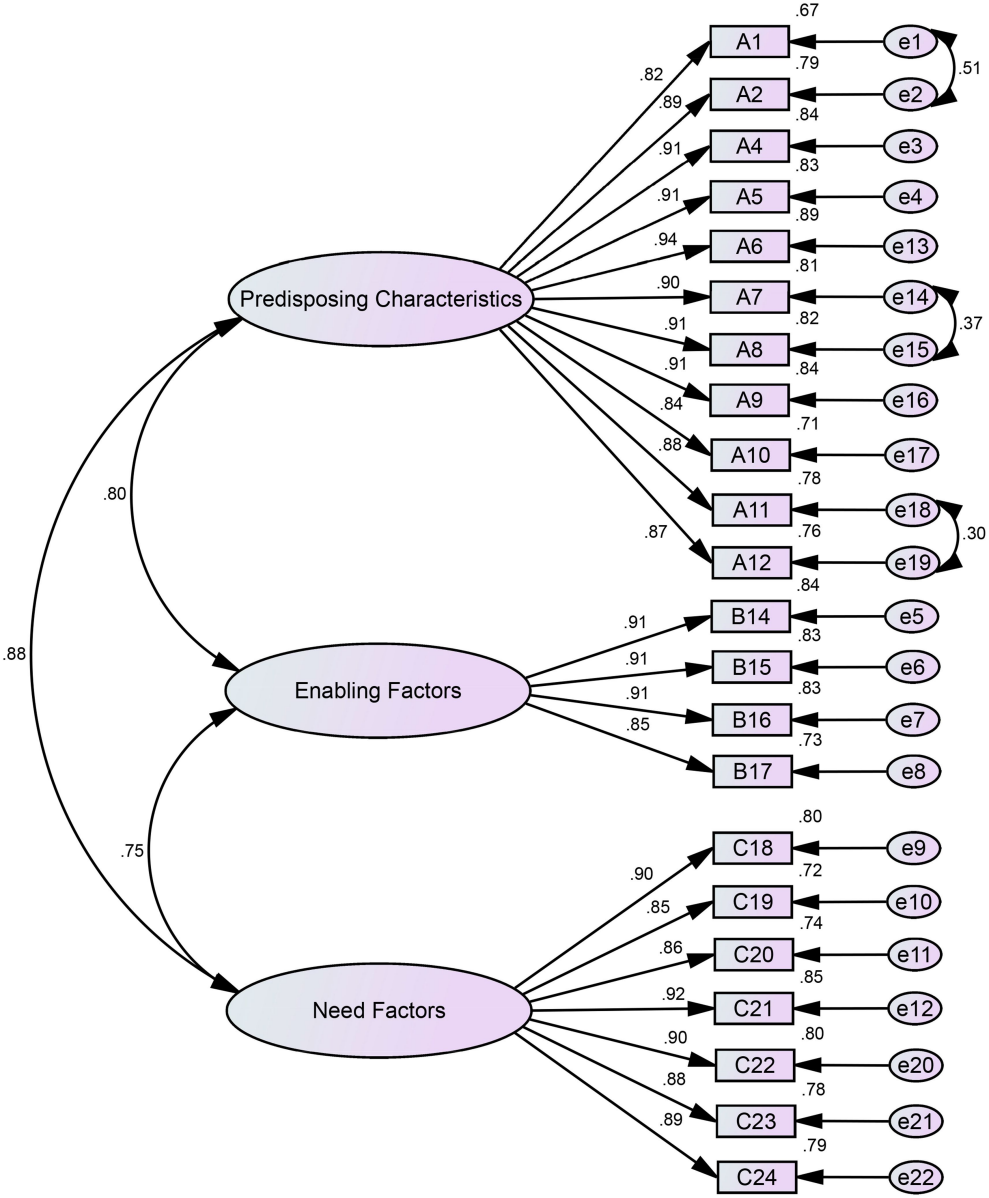
Modified results of the confirmatory factor analysis.

CFA was performed to confirm the three-factor structure of the scale. The initial model showed inadequate fit (*χ*^2^/*df* = 4.951, RMSEA = 0.092, CFI = 0.941, IFI = 0.941, TLI = 0.934). After model modifications, the fit indices improved significantly (*χ*^2^/*df* = 3.842, RMSEA = 0.078, CFI = 0.958, IFI = 0.958, TLI = 0.952), all reaching acceptable thresholds. The final model is shown in Figure 3. Detailed goodness-of-fit indices are presented Table 5.

**Table 5:** Goodness-of-fit indices for the confirmatory factor analysis models.

Model	*χ*^2^/*df*	RMSEA	CFI	IFI	TLI
Threshold value	<5	<0.08	>0.9	>0.9	>0.9
Initial model	4.951	0.092	0.941	0.941	0.934
Final model	3.842	0.078	0.958	0.958	0.952

χ^2^*/df*, Normed Chi-Square; RMSEA, root mean square error of approximation; CFI, comparative fit index; IFI, incremental fit index; TLI, Tucker-Lewis Index.

**Figure 4:**
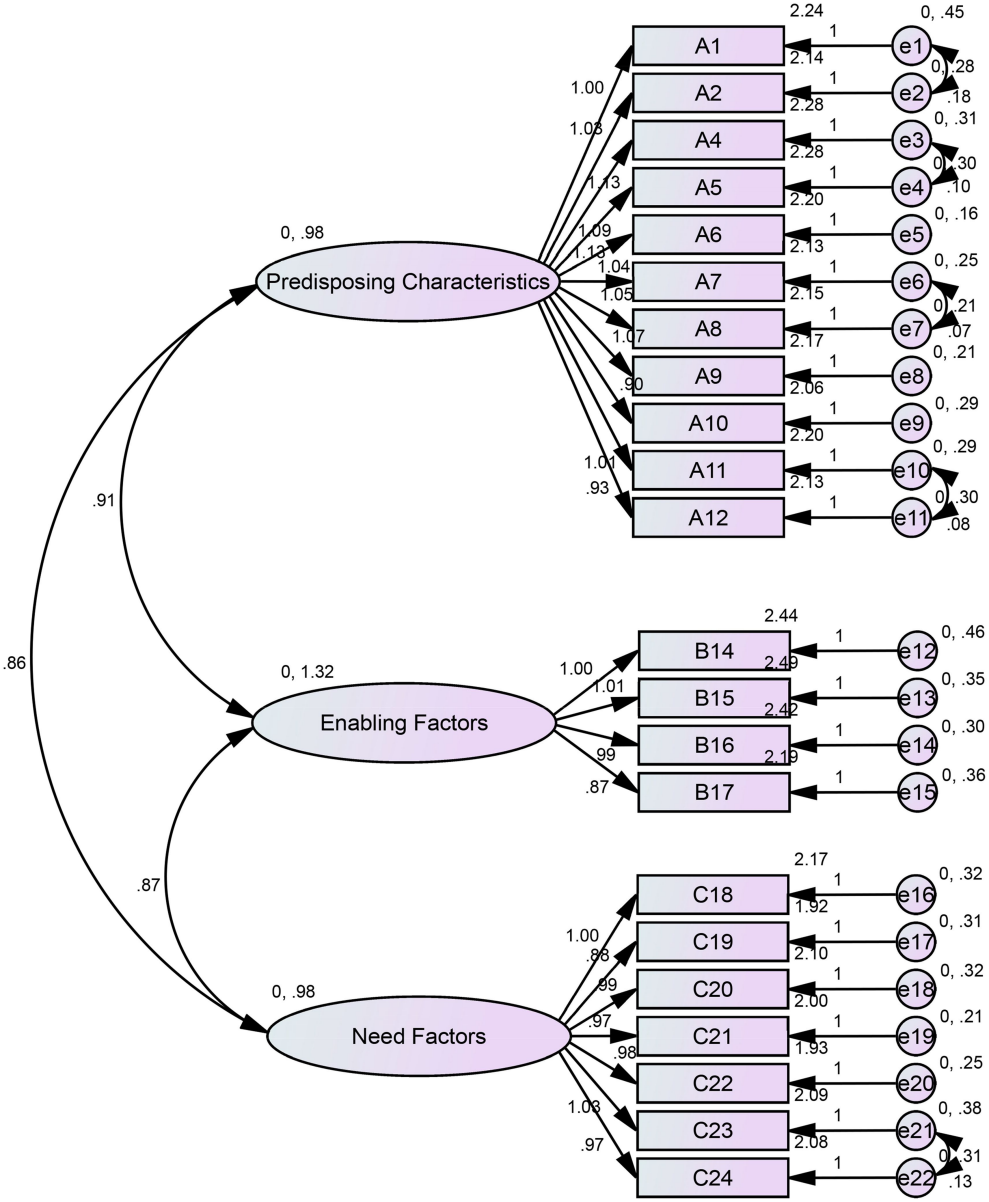
Modified results of the confirmatory factor analysis for the adolescent subgroup.

**Figure 5:**
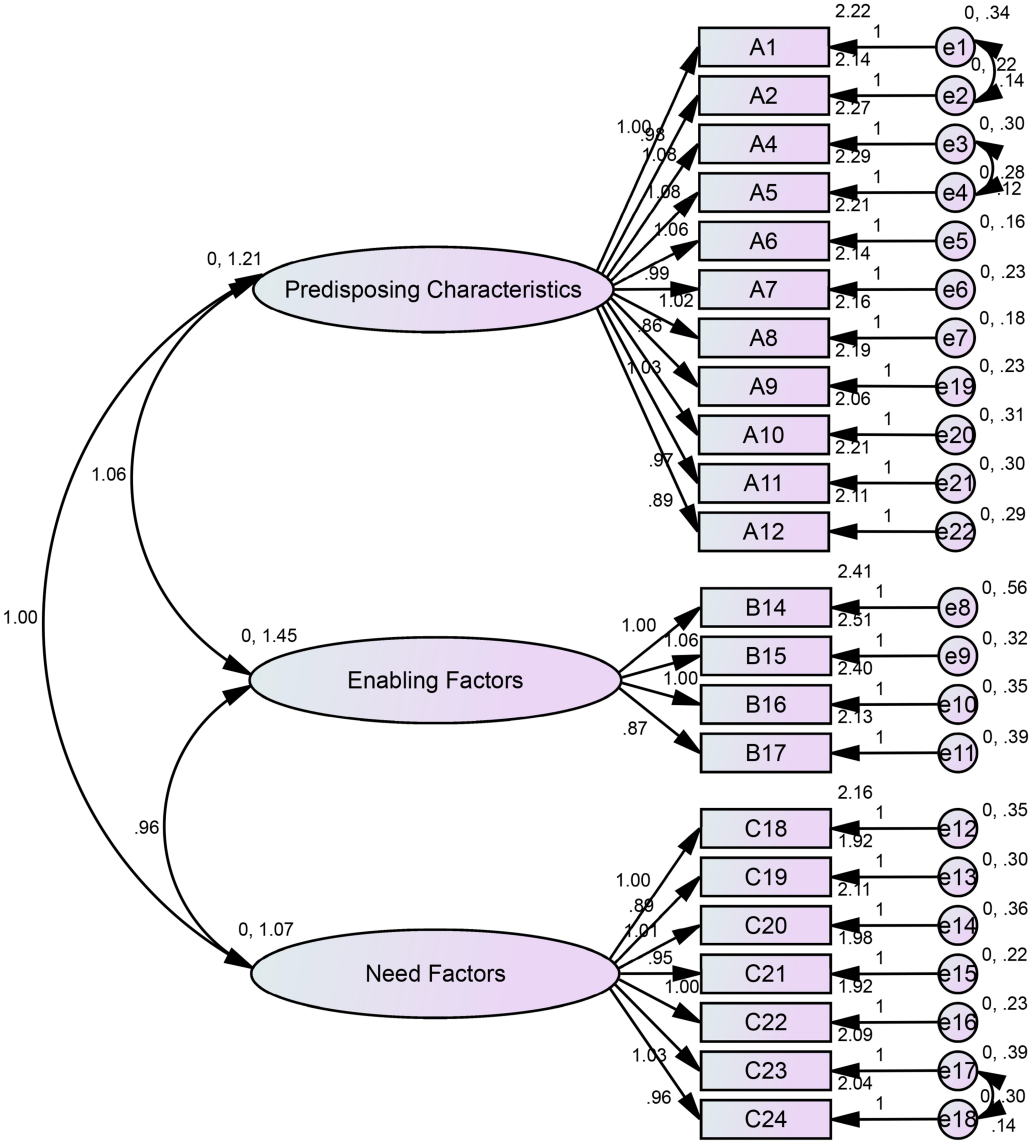
Modified results of the confirmatory factor analysis for the older adult subgroup.

To examine the stability of the factor structure across the two distinct populations, separate EFAs and CFAs were conducted for the adolescent (*n* = 465) and older adult (*n* = 290) subgroups. In both subgroups, EFA consistently extracted the same three-factor solution, with all items loading strongly (>0.50) on their intended factors. Subsequent CFA confirmed an acceptable model fit within each subgroup (Table 6). The final models for the adolescent and older adult subgroups are presented in Figures 4, 5 respectively.

**Table 6:** Goodness-of-fit indices for the confirmatory factor analysis models in adolescent and older adult subgroups.

Group	Sample size (*n*)	*χ*^2^/*df*	RMSEA	CFI	IFI	TLI
Threshold value	–	<5	<0.08	>0.9	>0.9	>0.9
Adolescents	465	3.984	0.073	0.961	0.962	0.956
Older adults	290	3.866	0.090	0.946	0.946	0.939

χ^2^*/df*, Normed Chi-Square; RMSEA, root mean square error of approximation; CFI, comparative fit index; IFI, incremental fit index; TLI, Tucker-Lewis Index.

### Differences in service needs between adolescents and older adults

Differences in scores on the Service Needs subscale between adolescents and older adults were analyzed using independent samples t-tests (Table 7). No statistically significant difference was found in the total Service Needs score between adolescents (Mean = 2.19, SD = 0.94) and older adults (Mean = 2.28, SD = 0.89) (*t* = −1.42, *p* = 0.156). At the item level, adolescents reported significantly higher needs for psychological counseling (A5) and for services accessed via digital platforms (A11). In contrast, older adults reported significantly higher needs for physical therapy services (C19), pharmacotherapy services (C20), and mental rehabilitation services (C22) (all *p* < 0.05). Within each group, psychological support from family (B14) and material support from family (B15) were among the items with the highest mean scores for both adolescents and older adults. For adolescents, psychological counseling (A5) was also a high-scoring item. For older adults, preventive guidance for mental health issues (A4) was among the highest-rated needs. The lowest mean scores for adolescents were observed for physical therapy services (C19) and mental rehabilitation services (C22). For older adults, the lowest mean scores were for services delivered in a “group training” format (A10) and psychological crisis intervention services (C23).

**Table 7:** Comparison of mental health service needs scores between adolescents and older adults.

Item (need level for the following services)	Adolescents (*n* = 465) (Mean ± SD)	Older adults (*n* = 290) (Mean ± SD)	Mean difference (95% CI)	*p*-value
Factor 1: predisposing characteristics				
A1 Mental health knowledge promotion	2.24 ± 1.20	2.32 ± 1.17	−0.08 (−0.25, 0.09)	0.362
A2 Diagnosis of mental disorders	2.14 ± 1.15	2.21 ± 1.15	−0.07 (−0.24, 0.10)	0.428
A4 Preventive guidance for mental health issues	2.28 ± 1.25	2.36 ± 1.15	−0.08 (−0.26, 0.10)	0.395
**A5 Psychological counseling**	**2.28 ± 1.24**	**2.21 ± 1.14**	**0.07 (−0.10, 0.24)**	0.019*
A6 Services provided directly by primary mental health institutions	2.20 ± 1.15	2.29 ± 1.13	−0.09 (−0.27, 0.09)	0.320
A7 Services provided by psychiatric departments of general hospitals / mental health specialty hospitals/private institutions cooperating with primary institutions	2.13 ± 1.14	2.25 ± 1.30	−0.12 (−0.31, 0.07)	0.206
A8 Continuation of treatment plans from superior hospitals by primary institutions	2.15 ± 1.13	2.27 ± 1.25	−0.12 (−0.31, 0.07)	0.213
A9 Services delivered in a “one-on-one” guidance format	2.17 ± 1.15	2.14 ± 1.21	0.03 (−0.15, 0.21)	0.735
A10 Services delivered in a “group training” format	2.06 ± 1.04	2.02 ± 1.09	0.04 (−0.13, 0.21)	0.649
**A11 Services accessed via “digital platforms”**	**2.20 ± 1.14**	**2.09 ± 1.06**	**0.11 (0.02, 0.20)**	0.012*
A12 Services accessed via “non-digital means”	2.13 ± 1.07	2.14 ± 1.03	−0.01 (−0.18, 0.16)	0.899
Factor 2: enabling factors				
B14 Psychological support from family during service receipt	2.44 ± 1.33	2.58 ± 1.21	−0.14 (−0.34, 0.06)	0.166
B15 Material support from family during service receipt	2.49 ± 1.30	2.53 ± 1.27	−0.04 (−0.24, 0.16)	0.691
B16 Psychological support from friends/colleagues during service receipt	2.42 ± 1.27	2.35 ± 1.16	0.07 (−0.12, 0.26)	0.457
B17 Material support from friends/colleagues during service receipt	2.19 ± 1.17	2.36 ± 1.20	−0.17 (−0.36, 0.02)	0.079
Factor 3: need factors				
C18 Psychotherapy services	2.17 ± 1.14	2.20 ± 1.23	−0.03 (−0.21, 0.15)	0.741
**C19 Physical therapy services**	**1.92 ± 1.03**	**2.17 ± 1.16**	**−0.25 (−0.41, −0.09)**	0.002*
**C20 Pharmacotherapy services**	**2.10 ± 1.14**	**2.35 ± 1.37**	**−0.25 (−0.41, −0.09)**	0.002*
C21 Follow-up management and guidance services	2.00 ± 1.06	2.14 ± 1.20	−0.14 (−0.31, 0.03)	0.102
**C22 Mental rehabilitation services**	**1.93 ± 1.09**	2.13 ± 1.22	**−0.20 (−0.37, −0.03)**	0.021*
C23 Psychological crisis intervention services	2.09 ± 1.19	2.00 ± 1.11	0.09 (−0.09, 0.27)	0.319
C24 Long-term psychological rehabilitation services post-crisis	2.08 ± 1.11	2.08 ± 1.12	0.00 (−0.18, 0.18)	0.996

Items follow the three-factor structure (see Table 4). Mean Difference (Adolescents − Older Adults) with 95% CI from independent samples t-tests. Significant items (*p* < 0.05) are in bold with an asterisk (*).

The final MHSDUQ contains 74 items organized according to the revised Andersen BMHSU framework. It comprises three theory-anchored sections: the Predisposing Characteristics section (22 items, presented as basic demographic information), the Service Needs subscale (22 items), and the Service Utilization and Evaluation module (29 items). The Service Utilization and Evaluation module is aligned structurally with the Service Needs subscale to permit direct need–utilization comparison, but it was not subjected to factor analysis because of limited data variability, as noted in the Limitations section.

## Discussion

### Scientific and normative development of the MHSDUQ

The global demand for mental health services is increasingly surpassing available resources (33), necessitating precise assessments of service needs and utilization to optimize resource allocation and inform policy development. Current research primarily addresses isolated assessment dimensions: the WMH-CIDI centers on psychiatric diagnosis (34), and Need for the Elderly (CANE) on individually perceived needs in specific domains (35). However, these tools do lack policy considerations, cultural factors, and population-specific demands. This study developed a culturally sensitive and psychometrically robust MHSDUQ, based on the framework of R-MUSDU. The revised framework incorporates two key constructs: perceived service evaluation and health service utilization, enhancing the understanding of help-seeking behaviors within specific sociocultural contexts. Addressing the mental health needs of adolescents and older adults is essential for promoting equitable and sustainable public health outcomes (36, 37). To ensure the sample’s representativeness, our sampling strategy deliberately encompassed participants from various health status categories, including healthy, sub-healthy, and clinically diagnosed individuals. This study employed a comprehensive mixed-methods design that surpasses conventional approaches reliant solely on literature reviews or semi-structured interviews. Focusing on high-risk populations, the research design integrated a tripartite approach for item generation, using bibliometric analysis (*n* = 4,864), policy document analysis (*n* = 8), and qualitative interviews (*n* = 17). Subsequent refinement of the instrument involved TEC (*n* = 18) and a pilot test (*n* = 60), followed by assessing its psychometric properties for reliability and validity in a large-scale survey (*n* = 755). Notably, the “Service Utilization and Evaluation” section was designed to directly correspond to the “Service Needs” section, enabling a comparative analysis of needs versus actual usage. During the psychometric validation, two items (A3 and A13) were removed from the Service Needs sub-scale, and the corresponding items in the utilization and evaluation section were also removed to preserve the structural integrity of the final questionnaire. The development of the MHSDUQ showcases methodological rigor and cultural adaptability, offering a valuable tool for accurately identifying mental health service needs among key populations and facilitating evidence-based resource allocation. This study exemplifies the scientific integrity achieved through the integration of theoretical construction with empirical validation.

### Excellent reliability and validity of the MHSDUQ

The study shows that the MHSDUQ has strong psychometric properties in evaluating mental health service needs among high-risk populations. The Service Needs subscale displayed high internal consistency, with Cronbach’s *α* of 0.975 and coefficients ranging from 0.863 to 0.968. These values significantly exceeded the acceptable internal consistency threshold of 0.70, indicating strong inter-item correlations and stable, consistent measurement of the intended latent construct. Split-half reliability analysis further supported this result (Spearman-Brown coefficient = 0.944). Item analysis confirmed strong discriminant validity, with all items showing critical ratios exceeding 3.000 and item-total correlations ranging from 0.765 to 0.914, indicating excellent item discrimination. The removal of item A3 due to its high correlation with item A4 (*r* = 0.859) enhanced the structural validity of the questionnaire. Construct validity was comprehensively assessed through exploratory factor analysis (EFA) and confirmatory factor analysis (CFA). EFA results revealed a clear and theoretically coherent three-factor structure (predisposing characteristics, enabling resources, and need factors), with 22 items accounting for 82.716% of the total variance and indicating a well-defined scale structure (38). Subsequent CFA confirmed this structure, with all fit indices (*χ*^2^/*df* = 3.842, RMSEA = 0.078, CFI = 0.958, TLI = 0.952, IFI = 0.958, TLI = 0.952) meeting or exceeding established thresholds for good model fit (39). The empirical reallocation of items B11 and B12 between factors demonstrates the balance between theoretical foundation and statistical optimization. These findings validate the theoretical applicability of Anderson’s model in the unique context and confirm the newly introduced concepts of “policy implementation” and “perceived service evaluation.” The MHSDUQ demonstrates a combination of high internal consistency, strong item discrimination, and a stable, well-fitting factorial structure.

### Practical applicability of the MHSDUQ

The findings from the MHSDUQ application reveal significant differences in mental health service needs between Chinese adolescents and older adults, identifying barriers specific to each population that can inform targeted policy and service improvements. Adolescents show a strong preference for accessible preventive services, like psychological counseling, but their help-seeking behavior is mainly hindered by attitudinal barriers such as stigma and privacy concerns. Conversely, older adults prioritize continuous community-based care but face structural obstacles, including perceived inadequate professional competence among primary care providers and challenges related to the digital divide. These findings align with existing literature on help-seeking behaviors in cultural contexts and underscore the urgent need for tailored interventions for distinct demographic groups. The development and validation of the MHSDUQ across multiple settings, including communities, schools, and healthcare institutions, demonstrate its utility for assessing needs at the grassroots level while maintaining broad applicability across diverse healthcare environments. This tool not only identifies unmet needs but also pinpoints specific obstacles to service utilization, thereby enabling more precise targeting of policy interventions and resource allocation. Aligning with the global goal of reducing mental health disparities and enhancing life course approaches to well-being, this study sets a new standard for understanding mental health service demands and offers a practical tool to support global public health efforts for vulnerable populations, including youth and the older adult. To address the identified obstacles, forthcoming endeavors should concentrate on enhancing primary care capacities and crafting age-specific digital solutions to enhance service availability. Additionally, the implementation of integrated care models that effectively link community-based and institutional services could notably enhance both the accessibility and acceptability of mental health assistance for these vulnerable groups. Consequently, the MHSDUQ not only functions as an evaluative instrument but also furnishes a comprehensive analytical framework for pinpointing crucial intervention junctures to fortify mental health service provision in China and analogous contexts.

## Conclusion

The MHSDUQ developed in this study is a theory-based tool developed and validated within a specific sociocultural and policy context. Grounded in Andersen’s behavioral model and incorporating dimensions of policy implementation and service perception evaluation, it effectively captures the complex interplay of environmental, individual, and service-related factors influencing mental health service utilization among key populations. The rigorous development process including literature bibliometric analysis, national policy review, qualitative interviews, and TEC to ensure strong content validity. High internal consistency (Cronbach’s *α* = 0.975) and satisfactory construct validity support its reliability and structural soundness. The MHSDUQ not only accurately assesses unmet needs and utilization patterns among Chinese adolescents and older adults but also provides practical evidence for policymakers and healthcare providers to develop targeted interventions and optimize mental health service delivery in China. Future research should further validate this tool across broader geographic and clinical populations to enhance its generalizability.

### Limitation

Certain limitations of this study warrant attention. First, due to considerations of patient cooperation and safety, the inclusion of patients with severe mental disorders accompanied by severe delusions or suicidal tendencies was relatively limited. To enhance the questionnaire’s applicability and generalizability, future studies will expand the sampling areas and increase the sample size. In-depth qualitative interviews will also be conducted to further analyze the health service needs of patients with severe mental disorders. Second, the generally low utilization rate of primary mental health services in China, which stems from insufficient resource allocation and infrastructure, resulted in limited variability in the “Service Utilization and Evaluation” data, thereby preventing factor analysis. Consequently, based on the initial research design, which aligned health service utilization and evaluation items with identified health service needs, the fourth dimension of the MHSDUQ, “Service Perception Assessment and Healthcare Utilization,” was developed and refined. Future work will involve a scientific analysis of the mental health service needs of the target population. The findings will be submitted to government departments to inform and optimize health policy allocation. Subsequently, a mixed-methods approach will be employed to revise the items pertaining to service utilization and evaluation.

## Data Availability

The raw data supporting the conclusions of this article will be made available by the authors, without undue reservation.

## Footnotes

1
https://www.gov.cn/zhengce/

2
http://www.nhc.gov.cn/

3
http://www.mca.gov.cn/

4
http://www.nhsa.gov.cn/

5
https://www.wjx.cn/
