# Supplementary material for: Development and validation of the mental health service demand and utilization questionnaire
Source: Front Public Health. 2026 Jan 12;13:1725107. doi: 10.3389/fpubh.2025.1725107 (PMC12833695; doi:10.3389/fpubh.2025.1725107)
Supplement: Supplementary file 1 [file Supplementary_file_1.docx]

**Additional file 1. Items Generated from Bibliometric Analysis**


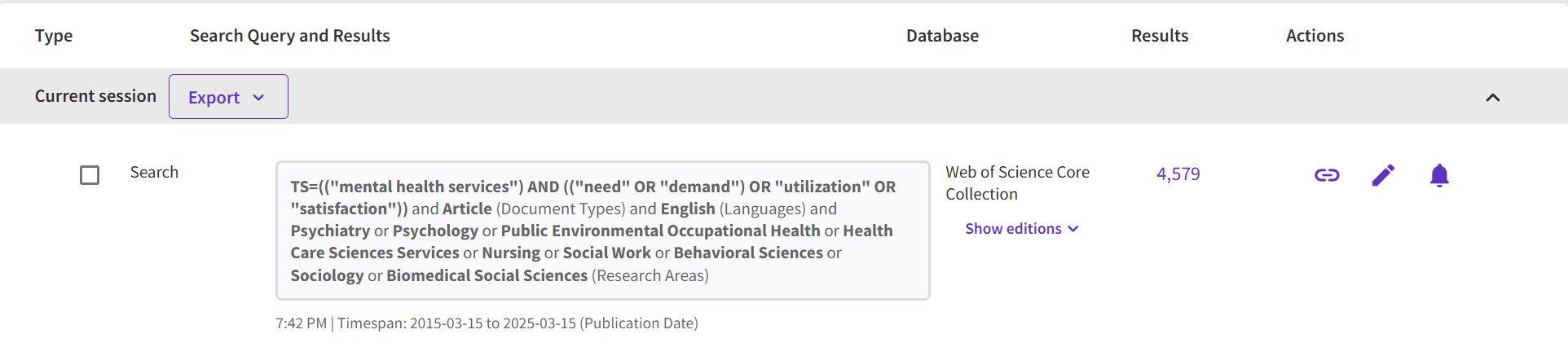


**Figure 1-1.** Search Strategy for the Bibliometric Analysis


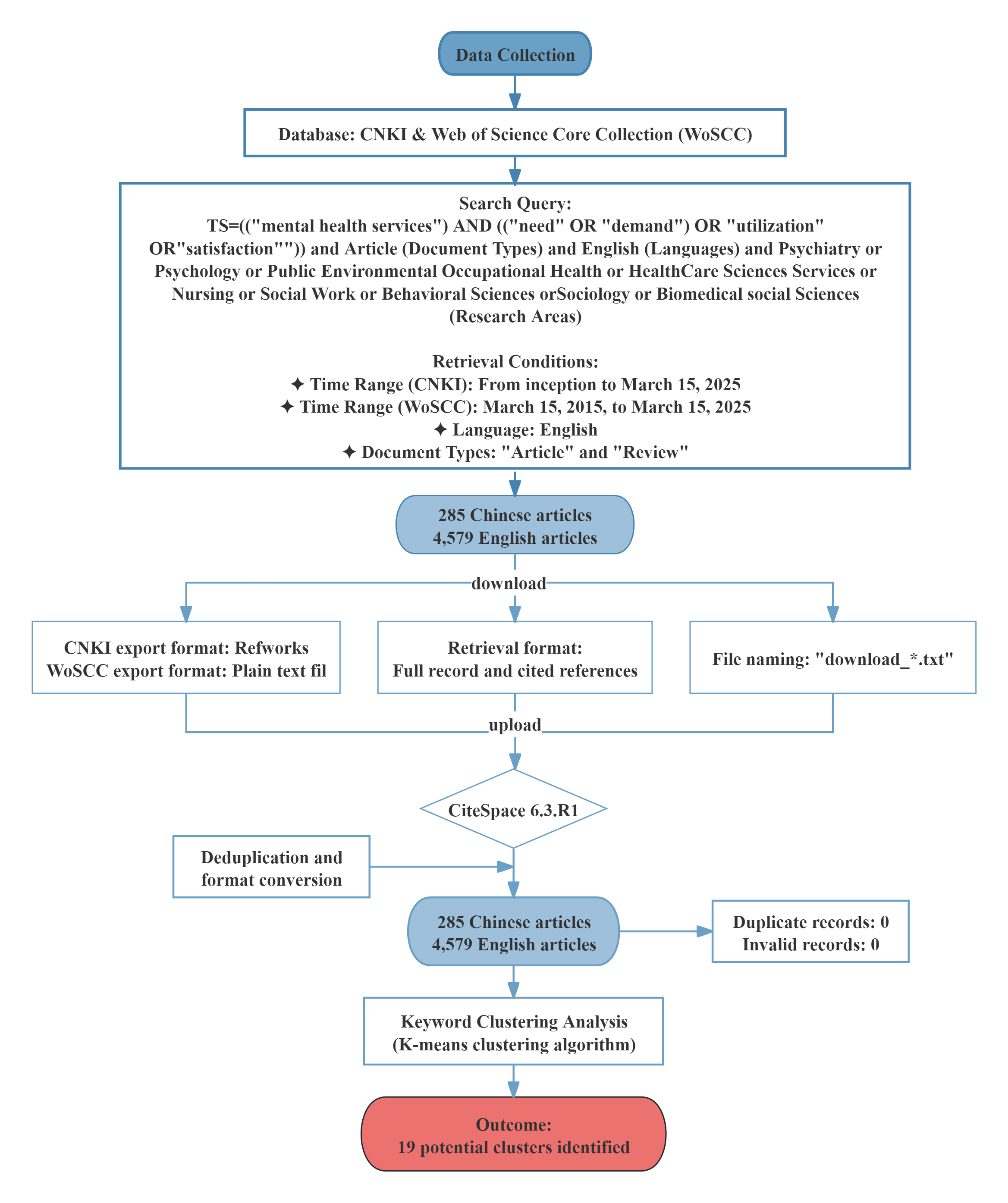


**Figure 1-2.** Workflow Diagram of Item Generation Based on Bibliometric Analysis

Keyword co-occurrence cluster analysis was conducted to identify core research themes within the mental health domain across two major databases. Analysis of the China National Knowledge Infrastructure (CNKI) database (**Figure 1-3**) revealed several central themes. Cluster #0, “Mental Health,” served as the core node, interconnected with #1 “Accessibility” (convenience of service access), #2 “Needs” (demand for mental health services), #3 “Service Utilization,” #4 “Community” (exploring community-based mental healthcare), and #5 “Mental Disorders” (research focused on the specific diseases). Collectively, these clusters signify a multifaceted exploration of China’s mental health service system, encompassing service accessibility, needs assessment, community applications, and clinical associations with mental disorders.

Cluster analysis of the Web of Science (WoS) Core Collection (**Figure 1-4**) centered on mental health services but with distinct emphases. Cluster #0, “Mental Health Services,” formed the core, linked to #1 “Randomized Controlled Trial,” #2 “Primary Care,” #3 “Mental Health,” #4 “Foster Care,” and #5 “Symptoms.” This suggests a significant international research emphasis on utilizing rigorous methodologies, such as randomized controlled trials, to examine the implementation of service systems, their integration within primary care, connections with social care systems (e.g., foster care), and outcomes at the symptom level.


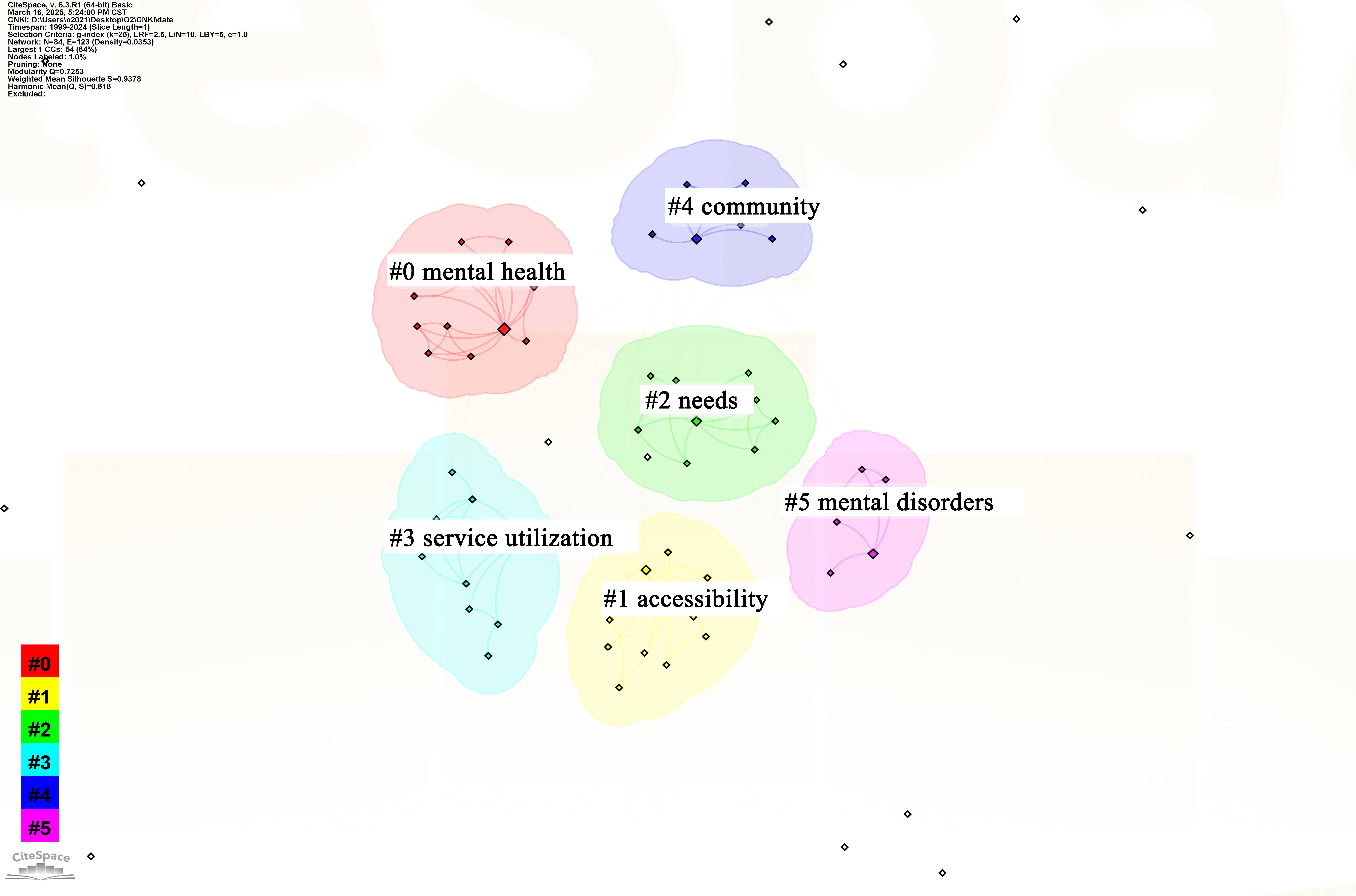


**Figure 1-3.** Keyword Clustering Map from the CNKI Database


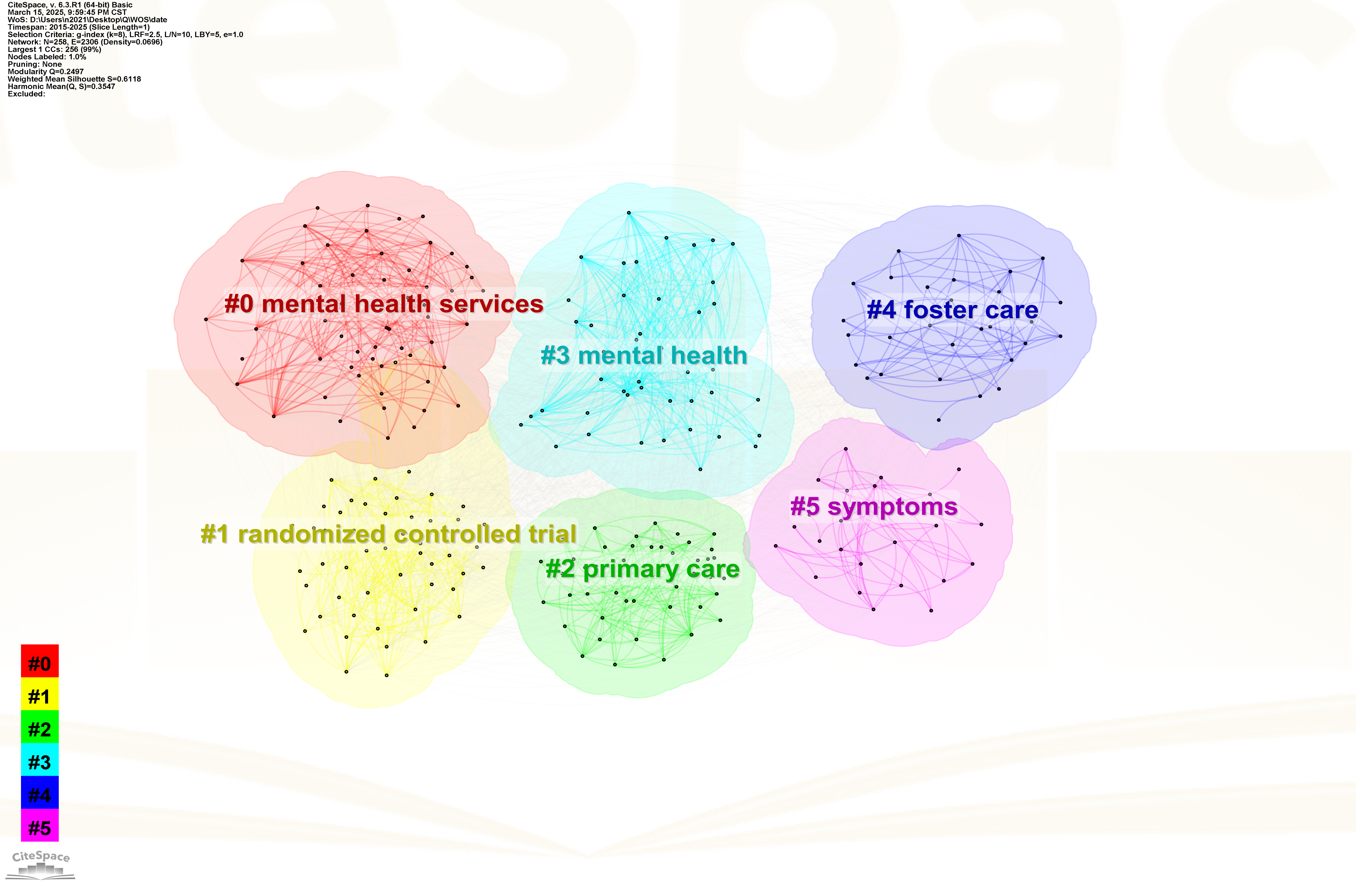


**Figure 1-4.** Keyword Clustering Map from the WoS Core Collection

**Table 1-1** and **Table 1-2** present the detailed keyword clusters and their contents from the CNKI and WoS Core Collection databases, respectively. The Chinese and English literature clusters share several commonalities: i) Both focus on core health service areas. CNKI-based research focused on “Mental Health Services,” “Needs,” and “Service Utilization” (e.g., #0, #2, #3), while WoS-based research focused on “Mental Health Services,” “Utilization,” and “Need” (e.g., #0, #3, #5), with both stressing the importance of constructing service systems and conducting needs assessments. ii) There is a significant emphasis on community-based care. Chinese literature emphasized “Rural Communities” and “Community Management” (#4, #5), whereas English literature examined “Community-based” and “Primary Care” (#0, #2), underscoring the vital role of communities as platforms for service delivery. iii) Both exhibit a shared concern for the needs of specific populations, such as “Detainees” and “Impoverished Individuals” (#2, #5) in Chinese research, and “Foster Care” and “African Immigrants” (#4, #5) in English research, reflecting a responsiveness to the service demands of vulnerable groups.

Differences between the clusters were primarily evident in three areas: i) Research perspectives. Chinese literature mainly focused on status surveys and policy practice, such as “Policy Implementation” and “Integrated Traditional Chinese and Western Medicine,” whereas English literature more frequently delved into the application and innovation of specific techniques or methods. ii) Application directions. Chinese literature often proposed localized innovative ideas based on national conditions, such as “Digital Services” and “AI-assisted Diagnosis and Treatment” (#1, #3). In contrast, English literature offered solutions from a more technological perspective, like “Digital Mental Health” and “E-mental Health” (#0, #1). iii) Contextual backgrounds. Chinese literature addressed China’s specific socio-economic context, including issues like mental health in rural areas (e.g., “Accessibility,” “Rural Communities”) and policy-driven work models (e.g., “Family Intervention”). Meanwhile, English literature engaged with prominent international topics, such as cross-cultural barriers and post-traumatic stress reactions. Additionally, CNKI clusters tended to describe service models, whereas WoS clusters provided more details on empirical research design and technical application. These differences highlight the distinct perspectives within domestic and WoS-based research. However, their complementarity in core themes like service needs and coverage of special populations offers a dual foundation of policy relevance and global perspective for the questionnaire design, collectively forming a multidimensional research landscape.

**Table 1-1.** Keyword clusters from the china national knowledge infrastructure (cnki) database

| **Cluster ID** | **Year** | **Top terms (LSI)** | **Top terms (log-likelihood ratio)** | **Terms (mutual information)** |
| --- | --- | --- | --- | --- |
| #0 | 2012 | Conditional discharge; Supported housing | Mental health; Mental health services; Conditional discharge; Supported housing; Different incomes | Conditional discharge; Supported housing; Different incomes; Status survey; Hong Kong |
| #1 | 2015 | Accessibility | Accessibility; Floor area ratio; Spatial layout; Equity; Zero Suicide | Accessibility; Floor Area Ratio; Spatial Layout; Equity; Zero Suicide |
| #2 | 2012 | Needs; Detainees | Needs; Detainees; Utilization; Rural communities; Zhongshan City | Detainees; Utilization; Rural communities; Zhongshan City; Intervention |
| #3 | 2017 | Service utilization; Composite International Diagnostic Interview (CIDI) | Service utilization; Mental disorders; Composite International Diagnostic Interview (CIDI); Cross-sectional survey; Influencing factors | Composite International Diagnostic Interview (CIDI); Cross-sectional survey; Influencing factors; Consultation rate; Medical institutions |
| #4 | 2009 | Chronic schizophrenia | Community; Chronic schizophrenia; Rehabilitation; Quality of life; Family intervention | Chronic schizophrenia; Rehabilitation; Quality of life; Family intervention; Investigation |
| #5 | 2009 | Mental disorders | Mental disorders; Service system; Community management; Rural areas; Social behavior | Service system; Community management; Rural areas; Social behavior; Mental disorders |

**Table 1-2.** Keyword clusters from the web of science (wos) core collection

| **Cluster ID** | **Year** | **Top terms(LSI)** | **Top terms (log-likelihood ratio)** | **Terms (mutual information)** |
| --- | --- | --- | --- | --- |
| #0 | 2017 | Mental health services; Qualitative analysis; Conduct problems; Young adulthood; Neurodevelopmental disorders \| Mental health; Digital mental health; E-mental health; Digital mental health interventions; Suicide attempt | Mental health services; Qualitative research; Peer support; Transition; Depression | Community-Based; African Immigrants; Center Rescue; Coal-Mine Fire; Clinical Outcome |
| #1 | 2017 | Mental health; Mental illness; Service-user perspective; Trauma-informed care; Digital health solution \| Mental health services; Mental health disorders; Sexual orientation; Gender identity; Trauma-informed care | Randomized controlled trial; Quality of life; Schizophrenia; Validity; People | Community-Based; African Immigrants; Center Rescue; Coal-Mine Fire; Clinical Outcome |
| #2 | 2018 | Mental health; Northeastern U; Psychiatric disorders; Care; Alcohol \| Mental health services; Co-occurring disorders; Qualitative methods; Case studies; Mental illness identity | substance use Primary care; United States; Substance use disorder; Major depression; Substance use | Center Rescue; CES-D; African American Ministers; Antisocial Personality Disorder |
| #3 | 2016 | Mental health; Mental illness; Service-user perspective; Public stigma; Data disaggregation \| Mental health services; Health services accessibility; Occupational health; Technology industry; Employee health | Mental health; Help-seeking; Perceived need; Social stigma; Stigma | Community-Based; African Immigrants; Center Rescue; Coal-Mine Fire; Clinical Outcome |
| #4 | 2019 | Mental health; Psychiatric services; Population survey; General practitioner; Siblings \| Mental health services; Posttraumatic stress disorder; Posttraumatic stress symptoms; Health service utilization; Primary care services | Foster care; Posttraumatic stress disorder; Child welfare; Maltreatment; School mental health | Caregiver Service Need; Attenuated Psychosis Syndrome; Agricultural; Behavioral Health; Child Behaviors |
| #5 | 2019 | Mental health; Mental health services; Sexual abuse; Siblings; African immigrants \| Mental healthcare; Service utilization; Siblings; African immigrants; Mothers | Symptoms; Domestic violence; Suicide; Postpartum depression; Primary care | Community-Based; African Immigrants; Coal-Mine Fire; Clinical Outcome; Cyber-Victimisation |

Based on the aforementioned keyword cluster analysis, a total of 19 potential attributes were initially identified, as detailed in **Table 1-3**.

**Table 1-3.** Potential attributes and corresponding levels identified through keyword clustering

| **No.** | **Potential Attribute** | **Corresponding Levels** |
| --- | --- | --- |
| ****1**** | ****Service Provider**** | public hospitals/community health center/private institutions/third-party social organizations |
| ****2**** | ****Service Type**** | community-based services/digital mental health services/peer support services/integrated care models/integrated traditional Chinese and Western medicine services |
| ****3**** | ****Service Accessibility**** | comprehensive coverage (urban-focused)/partial coverage (urban-rural disparities)/difficult to cover (remote areas) |
| ****4**** | ****Service Frequency**** | weekly/monthly/quarterly/as needed (irregular) |
| ****5**** | ****Service Duration per Session**** | ≤30 minutes/30-60 minutes/>60 minutes |
| ****6**** | ****Payment Method**** | public funding/health insurance reimbursement/partial out-of-pocket/full out-of-pocket |
| ****7**** | ****Service Continuity**** | fixed team for continuity of care/multi-agency collaboration/short-term or one-time service |
| ****8**** | ****Online Service Provision**** | no online services/basic online services (e.g., information push)/professional online services (e.g., remote consultation) |
| ****9**** | ****Integration of Traditional Chinese Medicine (TCM)**** | no TCM services/partial integration (e.g., adjunctive therapy)/full integration (integrated traditional chinese and western medicine) |
| ****10**** | ****Family Doctor Contracting**** | contracted/not contracted |
| ****11**** | ****Provider Professionalism**** | high professionalism (certified qualifications)/moderate professionalism/low professionalism |
| ****12**** | ****Coverage of Special Populations**** | adolescents/elderly/immigrants/trauma survivors/detainees/impoverished individuals/homeless individuals |
| ****13**** | ****Socio-demographic Factors**** | gender/income level/race/ethnicity/education level |
| ****14**** | ****Cultural Adaptation Measures**** | multilingual services/cultural sensitivity training/no specific measures |
| ****15**** | ****Type of Unmet Need**** | lack of psychological counseling/lack of crisis intervention/lack of rehabilitation management/lack of medication guidance |
| ****16**** | ****Health Management Tool Usage**** | mobile health applications/paper manuals/no tool usage |
| ****17**** | ****Technological Accessibility**** | digital device usage ability/internet coverage |
| ****18**** | ****Health Behavior Management Needs**** | exercise management/diet management/medication adherence |
| ****19**** | ****Psychological and Behavioral Factors**** | psychological distress (level of depression, anxiety)/suicidal ideation/health behaviors (exercise, diet management)/help-seeking behavior (willingness to actively seek help) |

***Note:*** Potential attributes are theoretical constructs or themes identified from the data. Their corresponding levels define operational expressions or value ranges, which were used to formulate the specific questionnaire items.
