# Supplementary material for: Development and validation of the mental health service demand and utilization questionnaire
Source: Front Public Health. 2026 Jan 12;13:1725107. doi: 10.3389/fpubh.2025.1725107 (PMC12833695; doi:10.3389/fpubh.2025.1725107)
Supplement: Supplementary file 2 [file Supplementary_file_2.docx]

**Additional file 2. Potential Attributes and Levels Expanded through Policy Document Analysis**

A systematic review of national-level policy documents was conducted to complement and extend the initial attributes derived from bibliometric analysis. Following screening, eight core policy documents were included for in-depth analysis (**Table 2-1**). These documents collectively address key strategic domains, including mental health service system development, promotion of digital mental health, protection of vulnerable populations, and integration of mental health services into medical insurance schemes. The policy directives therein provided a robust foundation for expanding the initial item pool and ensuring the questionnaire’s relevance to contemporary national priorities and implementation contexts.

**Table 2-1.** National-level policy documents related to mental health services

| **Document Code** | **Issuing Authority** | **Year** | **Document Title** |
| --- | --- | --- | --- |
| D1 | NHC* | 2015 | *National Mental Health Work Plan (2015-2020)* |
| D2 | State Council | 2016 | *“Healthy China 2030” Planning Outline* |
| D3 | NHC | 2022 | *Notice on Promoting 5G+ Healthcare Application Development* |
| D4 | State Council | 2022 | *14th Five-Year Plan for National Health (2021-2025)* |
| D5 | NHC | 2023 | *Guidelines on Further Improving the Healthcare Service System* |
| D6 | NHC | 2023 | *Management Measures for Resource Sharing and Referral in Community-based Rehabilitation Services for Mental Disorders* |
| D7 | NHSA* | 2024 | *Guidelines for Establishing Pricing Items for Psychotherapy Services (Trial)* |
| D8 | MCA* | 2025 | *Management Measures for Mental Health Welfare Institutions* |

***Note:*** *NHC, National Health Commission of the People’s Republic of China; SC, State Council of the People’s Republic of China; NHSA, National Healthcare Security Administration; MCA, Ministry of Civil Affairs of the People’s Republic of China.

Combined with the 19 potential attributes identified in Phase 1 (bibliometric analysis), the policy document analysis supplemented an additional 9 unique attributes (**Table 2-2**). These attributes reflect specific policy priorities and practical requirements that were not fully captured in the extant academic literature (e.g., policy awareness, enterprise-based service provision, and technical accessibility), thereby ensuring the questionnaire’s alignment with contemporary national mental health strategies and real-world service contexts.

**Table 2-2.** Potential attributes and corresponding levels with policy basis

| **No.** | **Potential Attribute** | **Corresponding Levels** | **Policy Source** |
| --- | --- | --- | --- |
| **1** | **Service Provider** | community health institutions/third-party social organizations | D4, D5 |
|  |  | enterprise mental health service departments | D4 |
|  |  | mental health welfare institutions | D8 |
| **2** | **Types of Services** | digital mental health services/integrated traditional Chinese and Western medicine services | D2, D4 |
|  |  | community-based rehabilitation services | D6 |
|  |  | psychiatric monitoring services | D7 |
|  |  | AI-assisted diagnosis and treatment | D4 |
| **3** | **Payment Methods** | special subsidies for mental health services | D1 |
|  |  | inclusion of mental health services in medical insurance catalogues | D7 |
| **4** | **Service Continuity** | multi-agency collaboration | D1 |
|  |  | standardized follow-up cycles for community rehabilitation | D6 |
| **5** | **Coverage of Special Groups** | adolescents/older adults | D1 |
|  |  | extremely impoverished individuals/homeless beggars | D8 |
| **6** | **Cultural Adaptation Measures** | bilingual mental health services in ethnic minority regions | D4 |
| **7** | **Use of Health Management Tools** | government-led AI mental health assessment platforms | D4 |
| **8** | **Technical Accessibility** | digital device usage ability/internet coverage | D3 |
| **9** | **Policy Awareness** | fully aware/mostly aware/moderately aware/slightly aware/completely unaware | D6 |

***Note:*** Potential attributes are theoretical constructs or themes identified from the data. Their corresponding levels define operational expressions or value ranges, which were used to formulate the specific questionnaire items.
