# Supplementary material for: Development and validation of the mental health service demand and utilization questionnaire
Source: Front Public Health. 2026 Jan 12;13:1725107. doi: 10.3389/fpubh.2025.1725107 (PMC12833695; doi:10.3389/fpubh.2025.1725107)
Supplement: Supplementary file 3 [file Supplementary_file_3.docx]

**Additional file 3. Items Generated from Qualitative Interviews**

To capture context-specific perspectives on mental health service demand and utilization (MHSDU) among adolescents and older adults—two key target populations—this study conducted in-depth semi-structured interviews. The interviews aimed to validate the attributes identified in Phase 1 (bibliometric analysis) and Phase 2 (policy analysis), while also uncovering novel, practice-relevant attributes not previously captured in the literature or policy documents.

From April 15 to May 10, 2025, a total of 17 in-depth interviews were conducted (see **Table 3-1** for interviewee characteristics and the interview guide), yielding 17 audio recordings with durations ranging from 5 to 20 minutes. The transcribed text amounted to approximately 30,000 Chinese characters.

The interview transcripts were analyzed using a grounded theory approach. The data were subsequently coded and categorized according to the “Theoretical Framework of Demand for and Utilization of Mental Health Services Adapted from Andersen’s Model.” The coding process involved three hierarchical levels: Level 1 (open coding) identified significant concepts from the raw data to form initial codes; Level 2 (axial coding) grouped these codes into main categories; and Level 3 (selective coding) synthesized the main categories into core theoretical categories.

This iterative process generated 80 open codes (A01-A80) with 201 reference points. A comprehensive coding framework comprising three levels was established (**Table 3-2**), resulting in 4 core categories, 11 main categories, and 80 initial categories. Representative excerpts from the original interview transcripts, illustrating the open coding process, are provided in **Table 3-3**.

**Table 3-1.** Interviewee characteristics, inclusion/exclusion criteria, and interview guide content

| **Interviewee Group** | **Inclusion/Exclusion Criteria** | **Interview Guide Content** |
| --- | --- | --- |
| Adolescents | As described in the main text | 1. Awareness of recent mental health policies. 2. Experience and satisfaction with services or perceived barriers. 3. Factors influencing service continuation or discontinuation. 4. Urgently needed need and unmet needs and suggestions for service improvement 5. Understanding of local mental health-related initiatives and the key areas for enhancement. |
| Older Adults |  |  |
| Experts in Health Economics Policy Research/Primary Health Care Management/ Clinical and Public Health Providers | Professional personnel in the health services field with at least one year of relevant work experience.Exclusion criteria:   1. Severe physical disabilities; 2. Severe mental disorders preventing cooperation. |  |

**Table 3-2.** Three-level coding framework derived from interview data

| **Core Category**  **(Reference Point)** | **Main Category**  **(Reference Point)** | **Initial Categories (Reference Point)** |
| --- | --- | --- |
| **C01 Contextual Characteristics(44)** | **B01 Policy Awareness & Dissemination (16)** | A01 Need for Enhanced Social Campaigns(8); A02 Need for Improved Awareness(8) |
|  | **B02 Structural Barriers(28)** | A03 Information Channel Barriers (9); A04 Geographic Accessibility Barriers (6); A05 Time Constraints (4); A06 Resource Scarcity Barriers (3); A07 Economic Constraints (2); A08 Information Asymmetry Barriers (1); A09 Policy Implementation Gaps (2); A10 Communication Barriers (1) |
| **C02 Individual Traits(48)** | **B03 Stigma & Social Prejudice(24)** | A11 Self-Stigma Cognitive Bias (7); A12 Family Cognitive Bias (7); A13 Social Stigma Bias (4); A14 Medication Misconceptions (1); A15 Openness to Experience Bias (1); A16 Expectation Discrepancy (2); A17 Self-Perception Bias (2) |
|  | **B04 Attitudes & Acceptance(7)** | A18 Passive Acceptance (3); A19 Pragmatic Acceptance (2); A20 Self-Reliance Preference (1); A21 Service Receptiveness (1) |
|  | **B05 Objective Support Conditions(4)** | A22 Geographic Accessibility (1); A23 Trust in Professionalism (1); A24 Interpersonal Support (1); A25 Economic Affordability (1) |
|  | **B06 Pressure & Support Systems(13)** | A26 Academic Pressure (1); A27 Family Support Compliance (1); A28 Parent-Child Dynamics (3); A29 Policy Compliance Factors (1); A30 Formalized Resources (3); A31 Supply-Demand Imbalance (1); A32 Doubts About Counseling Efficacy (1); A33 Insufficient Prioritization (1); A34 Group Symptomatology (1) |
| **C03 Service Perception Evaluation(34)** | **B07 Service Quality Evaluation(18)** | A35 Satisfaction with Professional Attitude (10); A36 Satisfaction with Facilities (4); A37 Satisfaction with Symptom Relief Timeliness (2); A38 Satisfaction with Staffing (1); A39 Satisfaction with Waiting Time (1) |
|  | **B08 Community Service Gaps(16)** | A40 Need for Community-Based Volunteer Services (1); A41 Need for Emotional Support Services (3);  A42 Need for Symptom Alleviation Services (3); A43 Need for Personalized Treatment (1); A44 Need for Group Therapy (1); A45 Need for Cognitive-Behavioral Interventions (1); A46 Need for Hospitalization (1); A47 Insufficient Community Education Resources (2); A48 Lack of Community Support Systems (1); A49 Shortage of Community Professionals (1); A50 Inaccessible Help-Seeking Channels (1) |
| **C04 Health Service Utilization(75)** | **B09 Educational System Service Needs(20)** | A51 Need for Optimized School Mental Health Curriculum (3); A52 Need for Student Psychological Attention (3); A53 Need for School Mental Health Campaigns (3); A54 Need for Improved School Professional Services (3); A55 Need for School Mental Health Lectures (2); A56 Need for School Psychological Assessments (2); A57 Need for School Mental Health Screenings (2); A58 Need for School-Based Counseling (2) |
|  | **B10 Service Optimization Measures(21)** | A59 Standardized Departmental Infrastructure (5); A60 Innovative Service Models (4); A61 Expansion of Professional Workforce (3); A62 Professional Training Programs (2); A63 Crisis Intervention Mechanisms (2); A64 Diversified Outreach Channels (2); A65 Decoupling of Medical & Counseling Services (1); A66 Follow-Up & Feedback Mechanisms (1); A67 Professional Psychological Counseling Services (1) |
|  | **B11 Compliance & Barrier Factors(34)** | A68 Perceived Service Efficacy (11); A69 Compliance Linked to Professional Competence (5); A70 Compliance Linked to Patient-Provider Rapport (3); A71 Compliance Linked to Treatment Outcome Expectations (3); A72 Time Conflict Barriers (3); A73 Institutional Trust Barriers (2); A74 Referral Behavior Barriers (1); A75 Privacy Concerns (1); A76 Self-Management Barriers (1); A77 Family Support Barriers (1); A78 Policy Support Barriers (1); A79 Comparative Treatment Experience (1); A80 Economic Burden Barriers (1) |

**Table 3-3.** Illustrative excerpts from original interview data with open codes

| **Main Category** | **Initial Categories** | **Verbatim Interview Excerpt** |
| --- | --- | --- |
| B01 Policy Awareness & Dissemination | A01 Need for Enhanced Social Campaigns | *“Like the elderly and left-behind children... um, I think some communities, some rural elderly still feel ashamed about mental health or emotional issues... they’re unwilling to come. Communities should really do more publicity.”* |
| B02 Structural Barriers | A04 Geographic Accessibility Barriers | *“But sometimes it feels too far, too much hassle... unwilling to come.”* |
| B03 Stigma & Social Prejudice | A13 Social Stigma Bias | *“I think families should pay attention, and... societal discrimination is serious.”* |
| B04 Attitudes & Acceptance | A18 Passive Acceptance | *“Counseling clients are mostly teens. And... some just don’t want to accept it, very passive, they’ll find all kinds of excuses not to come.”* |
| B05 Objective Support Conditions | A25 Economic Affordability | *“Who wouldn’t want something free? Everyone’s happy with state subsidies.”* |
| B06 Pressure & Support Systems | A28 Parent-Child Dynamics | *“Teens’ biggest problems are academic pressure, then school relationships, and... family parent-child issues.”* |
| B07 Service Quality Evaluation | A35 Satisfaction with Professional Attitude | *“This doctor’s temperament is good. Very gentle, very responsible.”* |
| B08 Community Service Gaps | A50 Inaccessible Help-Seeking Channels | *“Some communities... community mental health support is lacking. Hospitals can only meet patients’ basic needs, but for community or social problems... there’s no way to seek help.”* |
| B09 Educational System Service Needs | A56 Need for School Psychological Assessments | *“Teens’ schools, and... companies should regularly conduct national psychological evaluations, like sandplay or bar charts, to assess public mental health.”* |
| B10 Service Optimization Measures | A61 Expansion of Professional Workforce | *“Once or twice, you can’t achieve empathy, um... and it doesn’t solve anything. Also, our clinics... with 30-40 morning appointments, especially after sleep clinics opened... no time at all for proper analysis.”* |
| B11 Compliance & Barrier Factors | A68 Perceived Service Efficacy | *“Treatment effects are obvious, um... like after medication, does sleep improve? Mood improve? Once they feel improvements, they’re willing to come back later.”* |

Subsequent analysis of the semi-structured interview data provided robust validation for many of the potential attributes and levels identified in the preliminary literature review. Furthermore, the analysis revealed 10 additional potential attributes salient to the lived experiences of the target populations. The final expanded list of attributes and their corresponding levels, synthesized from the in-depth interviews, is presented in **Table 3-4**.

**Table 3-4.** Potential attributes and corresponding items expanded through qualitative interviews

| **Dimension** | **No.** | **Potential Attribute** | **Corresponding Levels** | **Source (Initial Codes)** |
| --- | --- | --- | --- | --- |
| Contextual Characteristics (Policy & Environmental Factors) | 1 | Policy Awareness & Dissemination | fully aware/partially aware/only heard of/completely unaware | Policy Cognition & Dissemination (A01-A02) |
|  | 2 | Structural Barriers | information channel inaccessibility/urban-rural resource disparity/policy implementation gaps/cultural communication barriers | Contextual Characteristics (A03-A10) |
| Individual Traits (Predisposing, Enabling, Need Factors) | 3 | Stigma | self-stigma (high/medium/low)/family cognitive bias (yes/no)/perceived social stigma (severe/moderate/none) | Socio-cultural Factors (A11-A17) |
|  | 4 | Service Acceptance Attitude (Health Beliefs) | active seeking/passive acceptance/pragmatic choice/service refusal | Psychological & Behavioral Factors (A18-A21) |
|  | 5 | Objective Support Conditions | family support strength/transportation convenience/health insurance coverage/community service accessibility | Enabling Resources (A22-A25) |
|  | 6 | Group-Specific Needs | adolescent academic pressure intervention needs/older adult loneliness mitigation needs/family relationship mediation needs/post-trauma psychological rehabilitation needs | Group-Specific Needs (A26-A34) |
| Service Perception Evaluation | 7 | Service Quality Evaluation | satisfaction with professional attitude/evaluation of hardware facilities/symptom relief timeliness/wait time reasonableness | Service Perception Evaluation (A35-A39) |
|  | 8 | Community Service Deficiencies | insufficient educational resources/shortage of professional manpower/lack of follow-up mechanisms/single help-seeking channel | Service Accessibility & Resource Allocation (A40-A50) |
| Health Service Utilization | 9 | Educational System Service Needs | optimized school mental health curriculum/regular psychological screening/on-site professional psychological counseling/regular mental health lectures | Mental health lectures Types of Service Needs (A51-A58) |
|  | 10 | Service Optimization Measures | standardized departmental infrastructure/digital service models/multidisciplinary team collaboration/improved crisis intervention mechanisms | Service Supply Innovation (A59-A67) |
|  | 11 | Compliance Barrier Factors | economic burden/time conflict/privacy concerns/doubts about efficacy/insufficient family support | Individual Behavioral Decision Mechanisms (A68-A80) |

***Note:*** Potential attributes are theoretical constructs derived from the qualitative interviews. Their corresponding levels define operational expressions or value ranges, which were used to formulate the specific questionnaire items.

Following this, a comprehensive synthesis was conducted to integrate findings from all three phases (bibliometric analysis, policy analysis, and qualitative interviews), resulting in a final consolidated framework of potential attributes and levels, as detailed in **Table 3-5**.

**Table 3-5.** potential attributes and corresponding levels synthesized from bibliometric analysis, policy analysis, and qualitative interviews

| **No.** | **Potential Attribute** | **Corresponding Levels** |
| --- | --- | --- |
| **1** | **Service Provider** | public hospitals/community health centers/private institutions/third-party social organizations/enterprise mental health service departments/mental health welfare institutions |
| **2** | **Service Type** | community-based services/digital mental health services/peer support services/integrated care models/integrated traditional Chinese and Western medicine services/community-based rehabilitation services/psychiatric monitoring services/AI-assisted diagnosis and treatment |
| **3** | **Service Accessibility** | comprehensive coverage (urban-focused)/partial coverage (urban-rural disparities)/difficult to cover (remote areas) |
| **4** | **Service Frequency** | weekly/monthly/quarterly/as needed (irregular) |
| **5** | **Service Duration per Session** | ≤30 minutes/30-60 minutes/>60 minutes |
| **6** | **Payment Method** | public funding/health insurance reimbursement/partial out-of-pocket/full out-of-pocket/special subsidies for mental health services/inclusion of mental health services in medical insurance catalogues |
| **7** | **Service Continuity** | fixed team for continuity of care/multi-agency collaboration/short-term or one-time service/standardized follow-up cycles for community rehabilitation |
| **8** | **Online Service Provision** | no online services/basic online services (e.g., information push)/professional online services (e.g., remote consultation) |
| **9** | **Integration of Traditional Chinese Medicine (TCM)** | no TCM services/partial integration (e.g., adjunctive therapy)/full integration (integrated traditional Chinese and Western medicine) |
| **10** | **Family Doctor Contracting** | contracted/not contracted |
| **11** | **Provider Professionalism** | high professionalism (certified qualifications)/moderate professionalism/low professionalism |
| **12** | **Coverage of Special Populations** | adolescents/elderly/immigrants/trauma survivors/detainees/impoverished individuals/homeless individuals/extremely impoverished individuals |
| **13** | **Socio-demographic Factors** | gender/income level/race/ethnicity/education level |
| **14** | **Cultural Adaptation Measures** | multilingual services/cultural sensitivity training/no specific measures; bilingual mental health services in ethnic minority regions |
| **15** | **Type of Unmet Need** | lack of psychological counseling/lack of crisis intervention/lack of rehabilitation management/lack of medication guidance |
| **16** | **Health Management Tool Usage** | mobile health applications/paper manuals/no tool usage; government-led AI mental health assessment platforms |
| **17** | **Technological Accessibility** | digital device usage ability/internet coverage |
| **18** | **Health Behavior Management Needs** | exercise management/diet management/medication adherence |
| **19** | **Psychological and Behavioral Factors** | psychological distress (level of depression, anxiety)/suicidal ideation/health behaviors (exercise, diet management)/help-seeking behavior (willingness to actively seek help) |
| **20** | **Policy Awareness** | fully aware/mostly aware/moderately aware/slightly aware/completely unaware; fully aware/partially aware/only heard of/completely unaware |
| **21** | **Structural Barriers** | information channel inaccessibility/urban-rural resource disparity/policy implementation gaps/cultural communication barriers |
| **22** | **Stigma** | self-stigma (high/medium/low)/family cognitive bias (yes/no)/perceived social stigma (severe/moderate/none) |
| **23** | **Service Acceptance Attitude (Health Beliefs)** | active seeking/passive acceptance/pragmatic choice/service refusal |
| **24** | **Objective Support Conditions** | family support strength/transportation convenience/health insurance coverage/community service accessibility |
| **25** | **Group-Specific Needs** | adolescent academic pressure intervention needs/older adult loneliness mitigation needs/family relationship mediation needs/post-trauma psychological rehabilitation needs |
| **26** | **Service Quality Evaluation** | satisfaction with professional attitude/evaluation of hardware facilities/symptom relief timeliness/wait time reasonableness |
| **27** | **Community Service Deficiencies** | insufficient educational resources/shortage of professional manpower/lack of follow-up mechanisms/single help-seeking channel |
| **28** | **Educational System Service Needs** | optimized school mental health curriculum/regular psychological screening/on-site professional psychological counseling/regular mental health lectures |
| **29** | **Service Optimization Measures** | standardized departmental infrastructure/digital service models/multidisciplinary team collaboration/improved crisis intervention mechanisms |
| **30** | **Compliance Barrier Factors** | economic burden/time conflict/privacy concerns/doubts about efficacy/insufficient family support |

***Note:*** Potential attributes are theoretical constructs or themes identified from the data. Their corresponding levels define operational expressions or value ranges, which were used to formulate the specific questionnaire items.
