# Supplementary material for: Development and validation of the mental health service demand and utilization questionnaire
Source: Front Public Health. 2026 Jan 12;13:1725107. doi: 10.3389/fpubh.2025.1725107 (PMC12833695; doi:10.3389/fpubh.2025.1725107)
Supplement: Supplementary file 4 [file Supplementary_file_4.docx]

**Table 4-1.** Composition of stakeholders in the transparent expert consultation (n = 23)

| **Panel group** | **Sub-group** | **Education Level** | **Professional Field/ Position** | **Working Experience(Years)** | **Number of Participants** |
| --- | --- | --- | --- | --- | --- |
| Experts  (n = 6) | Health Policy | Ph.D. | Health Policy, Policy Research | 15 | 1 |
|  | Health Policy | Ph.D. | Health Service Management, Policy Research | 14 | 1 |
|  | Health Policy | M.D. | Health Systems Research, Primary Care | 10 | 1 |
|  | Primary Care Manager | M.D. | Primary Health Care Management | 22 | 1 |
|  | Health Economics Expert | Ph.D. | Health Economics, Policy Analysis | 12 | 1 |
|  | Primary Care Administrator | M.D. | Primary Health Care Administration | 18 | 1 |
| Clinical and public-health providers  (n = 5) | Psychiatrist | M.D. | Psychiatry/Chief Physician | 35 | 1 |
|  | Psychiatrist | M.D. | Psychiatry/Associate Chief Physician | 20 | 1 |
|  | General Practitioner | M.D. | General Practice/Attending Physician | 15 | 1 |
|  | General Practitioner | M.D. | Family Medicine/Physician | 8 | 1 |
|  | Public Health Physician | M.D. | Preventive Medicine/Public Health Specialist | 25 | 1 |
| Service users  (n = 6) | Healthy Adolescent | Secondary School | — | — | 1 |
|  | Adolescent Sub-health Individual | Secondary School | — | — | 1 |
|  | Adolescent Patient | Secondary School | — | — | 1 |
|  | Healthy Older Adult | Primary School to University and Above | — | — | 1 |
|  | Older Adult Sub-health Individual | Primary School to University and Above | — | — | 1 |
|  | Older Adult Patient | Primary School to University and Above | — | — | 1 |
| Guardians & caregivers(n = 6) | — | Primary School to University and Above | — | — | 6 |

***Note:*** M.D., Master Degree; Ph.D., Doctor of Philosophy in Medicine; Interquartile range. Scores ranged from 1 to 5.

**Table 4-2.** Revision of questionnaire items based on transparent expert consultation recommendations

| **Recommendations from Stakeholders** | **Median (IQR)^a^** | **Rationale and Revision Content** |
| --- | --- | --- |
| **Theoretical and Scientific Recommendations** | | |
| Suggest adding an item on “family history of mental disorders” to control for genetic confounding factors. | 4.5(0.8) | **Rationale:** Psychiatric epidemiology recognizes family history as a significant non-modifiable risk factor for mental disorders.  **Revision:** Added a binary (yes/no) item: “Have any of your first-degree relatives within three generations been diagnosed with a mental disorder?” followed by a multiple-choice list of common disorders (e.g., depression, schizophrenia, anxiety disorders). |
| Recommend distinguishing between “primary care institutions” and “non-primary care institutions” within the “Service Needs” dimension to reflect differences in service accessibility. | 4.2(1.1) | **Rationale:** Health services research emphasizes the structural and functional differences between primary and specialized care settings, which directly affect service availability and referral pathways.  **Revision:** The “Service Needs” dimension was subdivided into two subscales: “Needs for Primary Care Services” and “Needs for Specialized Mental Health Services,” with parallel items to capture setting-specific barriers. |
| Recommend reorganizing the options for “healthcare payment methods.” | 4.3(0.7) | **Rationale:** In line with the 2024 integrated medical insurance reform by the National Healthcare Security Administration, the former urban and rural insurance schemes have been unified.  **Revision:** The option “New Rural Cooperative Medical Scheme” was removed and merged into “Urban and Rural Resident Basic Medical Insurance” to reflect current policy. |
| **Practical Applicability Recommendations** | | |
| Recommend clarifying standards for “workforce allocation,” including “duration of transition training” and “competency of general practitioners.” | 4.0(0.9) | **Rationale:** National guidelines on primary mental health services (NHCC, 2023) specify minimum training requirements and core competencies for general practitioners delivering mental healthcare.  **Revision:** Added two items: “Have you completed ≥10 hours of provincial-level mental health transition training?” and “Are you able to independently conduct mental health screening, initial assessment, and patient referral?” |
| Recommend providing examples for “digital platforms” to avoid misinterpretation. | 3.6(1.2) | **Rationale:** Health literacy frameworks indicate that technical terms such as “digital platforms” may be misinterpreted by respondents with varying levels of familiarity with e-health.  **Revision:** The item was rephrased to: “Have you used digital platforms (e.g., mental health mobile apps, online counseling platforms, telepsychiatry) to access mental health services?” |
| Recommend finer income brackets to avoid selection bias. | 4.1(0.6) | **Rationale:** Regional socioeconomic data (e.g., average pension levels in Hebei Province) indicate that coarse income categories may mask economic disparities affecting mental health service utilization.  **Revision:** Monthly personal income brackets were refined to: “140–399 USD,” “400–699 USD,” and “≥700 USD”. |
| Recommend adding “cultural adaptation support” to accommodate multicultural groups. | 4.0(1.0) | **Rationale:** Cultural competence in mental health services is essential for engaging diverse populations, particularly ethnic minorities and migrant groups.  **Revision:** A new item was introduced: “Do you require mental health services that accommodate specific cultural, linguistic, or customary needs?” |
| **Policy Alignment Recommendations** | | |
| Recommend adding evaluation items for “policy promotion efforts" and "policy awareness.” | 4.3(1.0) | **Rationale:** The National Social Psychological Service System Construction initiative highlights policy awareness and dissemination as key to successful implementation.  **Revision:** Two items were added under service evaluation: “How would you rate the government’s efforts to promote mental health policies?” and “How well-informed are you about existing mental health policies?” |
| Recommend including “health insurance reimbursement ratio” as a sub-item under “economic barriers,” in response to health payment reform policies. | 4.4(0.8) | **Rationale:** Health economic studies identify inadequate insurance reimbursement as a major financial barrier to mental health service use.  **Revision:** Under “economic barriers,” a new option was included: “Insufficient coverage or low reimbursement rate by health insurance.” |
| Recommend incorporating items related to “family doctor contracting,” aligning with national family doctor contract service policies. | 4.2(0.9) | **Rationale:** The Family Doctor Contract Service Policy is a cornerstone of China’s primary care system, and enrollment status may influence access to and continuity of mental healthcare.  **Revision:** A dichotomous item (yes/no) was added to the basic information section: “Are you currently enrolled in a family doctor contract service program?” |

***Note:*** IQR, interquartile range. Scores ranged from 1 to 5.

**Table 4-3.** Feedback from pilot survey respondents and corresponding revisions

| **Recommendations from Respondents** | **Rationale and Revision Content** |
| --- | --- |
| **Clarity of Item Wording** | |
| Suggest adding explanatory notes for technical terms such as “psychotherapy” and “physical therapy.” | **Rationale:** Health communication principles emphasize the need to simplify professional terminology for better public understanding.  **Revision:** Added a “Note” box below relevant items to explain term definitions and common methods. |
| Recommend providing an operational definition for “service continuity.” | **Rationale:** Questionnaire design standards require clear operational definitions for all constructs.  **Revision:** Specified the criteria as: “continuous and stable (≥3 months),” “intermittent (with interruptions ≥2 times),” and “single service.” |
| **Completeness of Response Options** | |
| Suggest including “unaware of service content” as a reason for not using services. | **Rationale:** The pilot survey found that 26.7% of non-users attributed their lack of service use to being “unaware of service content.”  **Revision:** Added “unaware of service content” as an option under “reasons for non-use.” |
| Recommend expanding “types of services” to include “psychological crisis intervention” and “long-term psychological rehabilitation.” | **Rationale:** Mental health service guidelines highlight crisis intervention as a core component of care.  **Revision:** Added “psychological crisis intervention” and “long-term psychological rehabilitation” to the “types of services” options. |
| Suggest adding “student” as an occupational category to improve relevance for adolescents. | **Rationale:** The target population characteristics indicate that most adolescents are students.  **Revision:** Added “student” as an option under “occupation.” |
| **Respondent Burden and Logical Flow** | |
| Questionnaire length was excessive (68 items in the second version), with completion time exceeding 40 minutes. | **Rationale:** Questionnaire design principles recommend controlling response burden, with ideal completion time ≤30 minutes.  **Revision:** Deleted 12 redundant items to shorten the questionnaire. |
| Recommend merging “service utilization” and “service evaluation” into one module. | **Rationale:** The theoretical framework of this study posits that service utilization and evaluation belong to the same dimension.  **Revision:** Combined into a single “Service Utilization and Evaluation” module for improved structural coherence. |
| Lack of an entry for “guardian contact information” for minors raises ethical concerns. | **Rationale:** Medical ethics require special protections for minors in research.  **Revision:** Added a note under “contact information”: “For minors, please provide guardian contact details.” |
| Suggest optimizing skip logic to reduce invalid responses. | **Rationale:** The pilot survey identified unclear skip logic leading to invalid answers.  **Revision:** Added skip prompts, e.g., “If you have not used the service, please skip to Question 25.” |
